# *Bothrops* venom-induced hemostasis disorders in the rat: Between Scylla and Charybdis

**DOI:** 10.1371/journal.pntd.0011786

**Published:** 2023-11-27

**Authors:** Sébastien Larréché, Lucie Chevillard, Georges Jourdi, Simon Mathé, Aurélie Servonnet, Bérangère S. Joly, Virginie Siguret, Jean-Philippe Chippaux, Bruno Mégarbane

**Affiliations:** 1 Université Paris Cité, Inserm UMRS-1144, Paris, France; 2 Department of Medical Biology, Bégin Military Teaching Hospital, Saint-Mandé, France; 3 Université Paris Cité, Inserm UMRS-1140, Innovative Therapies in Hemostasis, Paris, France; 4 Department of Biological Hematology, Lariboisière Hospital, APHP, Paris, France; 5 Unité analyses biologiques, Institut de Recherche Biomédicale des Armées, Brétigny-sur-Orge, France; 6 Université Paris Cité, EA3518, Institut de Recherche Saint-Louis, Paris, France; 7 Université Paris Cité, Research Institute for Development, Mother, and Child in Tropical Environment: Pathogens, Health system and Epidemiological transition, Paris, France; 8 Department of Medical and Toxicological Critical Care, Lariboisière Hospital, Federation of Toxicology, APHP, Paris, France; Instituto Butantan, BRAZIL

## Abstract

Hemostasis impairment represents the most threatening consequence of Viperidae envenoming, notably with *Bothrops* genus. In the French departments of America, *B*. *atrox* envenomation in French Guiana may lead to bleeding while *B*. *lanceolatus* envenomation in Martinique to thrombosis. Bleeding related to *B*. *atrox* envenomation is attributed to vascular damage mediated by venom metalloproteinases and blood uncoagulable state resulting from thrombocytopenia and consumptive coagulopathy. Thrombosis related to *B*. *lanceolatus* envenomation are poorly understood. We aimed to compare the effects of *B*. *atrox* and *B*. *lanceolatus* venoms in the rat to identify the determinants of the hemorrhagic *versus* thrombotic complications. Viscoelastometry (ROTEM), platelet count, plasma fibrinogen, thrombin generation assay, fibrinography, endothelial (von Willebrand factor, ADAMTS13 activity, ICAM-1, and soluble E-selectin), and inflammatory biomarkers (IL-1β, IL-6, TNF-α, MCP-1, and PAI-1) were determined in blood samples obtained at H3, H6, and H24 after the subcutaneous venom *versus* saline injection. In comparison to the control, initial fibrinogen consumption was observed with the two venoms while thrombocytopenia and reduction in the clot amplitude only with *B*. *atrox* venom. Moreover, we showed an increase in thrombin generation at H3 with the two venoms, an increase in fibrin generation accompanied with hyperfibrinogenemia at H24 and an increase in inflammatory biomarkers with *B*. *lanceolatus* venom. No endothelial damage was found with the two venoms. To conclude, our data support two-sided hemostasis complications in *Bothrops* envenoming with an initial risk of hemorrhage related to platelet consumption and hypocoagulability followed by an increased risk of thrombosis promoted by the activated inflammatory response and rapid-onset fibrinogen restoration.

## Author summary

*Bothrops* venoms contain toxins targeting hemostasis, a physiological process aiming stopping bleeding in case of injury. Envenomation by *B*. *atrox* in French Guiana leads to hemorrhage while envenomation by *B*. *lanceolatus* in Martinique results in thrombosis. To understand the mechanisms involved in these opposite manifestations related to the two venoms, we designed a comparative rat study of hemostasis based on a comprehensive approach including blood measurement of initiation time and size of the clot, platelet count, plasma fibrinogen, thrombin and fibrin generation, and various inflammatory and endothelial biomarkers. We showed that *B*. *atrox* venom induces more marked platelet and fibrinogen consumption, promoting the risk of hemorrhage, while *B*. *lanceolatus* venom induces an initial fibrinogen consumption followed by a secondary increase in combination to an enhanced inflammatory reaction resulting in increased thrombosis risk. Interestingly, the two venoms exhibited no endothelial toxicity.

## Introduction

Hemostasis aims to stop bleeding in the event of vessel injury. Its three distinct phases, i.e., primary hemostasis, coagulation, and fibrinolysis, are closely linked [1]. In the primary hemostasis the injured vessel wall recruits platelets by activating von Willebrand factor (VWF), a multimeric glycoprotein; thereafter, the activated platelets recruit additional platelets to form the platelet aggregate [2]. Initiation of the coagulation process is the consequence of the exposure of tissue factor (TF) and leads to thrombin generation [3]. Fibrinogen cleavage mediated by thrombin and its subsequent polymerization to form fibrin strands provide the network required for the effective clot formation [4]. Finally fibrin clot is removed from the repaired blood vessels by fibrinolysis [5]. ADAMTS13 (*a disintegrin and metalloprotease with thromboSpondin type 1 repeats*, *member 13*) cleaves specifically VWF that unfolds under shear stress, to reduce the size of VWF polymers in circulation [6].

Hemorrhagic syndrome associated with the absence of blood coagulation are one of the main characteristics of Viperidae envenomation [7,8]. Other manifestations include pain, edema, blistering, dermo- and myonecrosis, hypovolemia, cardiovascular collapse, acute kidney injury, and more rarely thrombosis and neurotoxicity [9]. Viperidae are present in all continents except Oceania and *Bothrops* genus is mostly involved in Latin America [10]. Toxins from *Bothrops* venoms targeting hemostasis exhibit highly diverse enzymatic (snake venom metalloproteinases (SVMPs), snake venom serine proteases (SVSPs), phospholipases A_2_ (PLA_2_s) and L-amino acid oxidases) and non-enzymatic proteins (disintegrins and C-type lectin proteins) [11].

In the French departments of America, *B*. *atrox* is the predominant species involved in envenomation in French Guiana [12,13], whereas *B*. *lanceolatus* is the only venomous snake in Martinique, where it is endemic [14]. While these two species are very close phylogenetically (*B*. *lanceolatus* being included in *B*. *atrox* group [15]), they are responsible for opposite manifestations.

*B*. *atrox* bite may result in a typical Viperidae envenomation with the risk of local and systemic bleeding like other *Bothrops* such as *B*. *asper* and *B*. *jararaca* [16–20]. Systemic bleeding is associated with a higher death risk [21,22]. Bleeding is related to SVMP-degradation of basement membrane [23–26] and the subsequent disruption of endothelial cell integrity due to the enhanced hydrostatic pressure and tangential shear stress [27]. Thrombocytopenia and unclottable blood on admission are independently associated with systemic bleedings in *B*. *atrox-* and *B*. *jararaca*-envenomated patients admitted to the hospital [22,28,29]. SVMPs are able to clot citrated plasma by activating prothrombin and/or factor X [30–32]. Thrombocytin, a SVSP isolated from *B*. *atrox* venom, activates factors V, VIII, and XIII [33,34]. Venom-induced activation of clotting factors generates endogenous thrombin thus consuming fibrinogen [35]. Defibrinogenation also depends on the action of thrombin-like enzymes (TLE), which directly cleave fibrinogen in fibrin and induce *in vitro* clotting of fibrinogen [36]. Non-coagulant proteinases exhibiting fibrin(ogen)olytic activity additionally contribute to fibrinogen consumption without converting it to fibrin [37].

By contrast, *B*. *lanceolatus* envenomation is unusually associated with bleeding and absence of coagulability, but may be complicated by multiple systemic infarctions up to 48 h after the snakebite, even in case of moderate envenomation [38,39]. Thrombosis occur in cerebral, myocardial or pulmonary vessels and may be fatal or lead to major functional sequelae in the absence of antivenom administration [39,40]. The exact mechanism of thrombosis in *B*. *lanceolatus* envenomation is poorly understood. Despite venom-induced thrombocytopenia in mice, *B*. *lanceolatus* venom induced no direct effects on platelet aggregation in human platelet-rich plasma [41,42]. *B*. *lanceolatus* venom dose-dependently clotted purified human fibrinogen, indicating the involvement of a thrombin-like enzyme. However, it is devoid of defibrinating activity after intravenous injection in mice [41,43,44]. Interestingly, this venom was unable to clot citrated plasma in the first studies [41,43,44], suggesting the absence of procoagulant activity until new studies adding calcium to the plasma showed effects on coagulation [45,46]. Consistent, a fatal *B*. *lanceolatus* envenomation case with diffuse thrombotic microangiopathy (TMA) causing multiple cerebral, myocardial, and mesenteric infarctions on autopsy supported the possible onset of venom-induced endotheliopathy [47].

Surprisingly, venom composition of these two *Bothrops* species are relatively similar with comparable activities on coagulation *in vitro* in the whole blood and plasma [48]. However, *in vitro* assays have limitations as they only offer a view of the immediate venom-induced effects whereas envenomation is a complex dynamic process. Crude venoms exhibit strong dose-dependent procoagulant effects *in vitro* [49–51], while *in vivo* consumption of clotting factors result in blood hypocoagulability. To observe the time-dependent toxicity of the various enzymes present in Viperidae venoms, it is necessary to investigate the time-course of hemostasis disorders *in vivo* [52–55]. Recent global hemostasis assays, which have revolutionized the approach of hemorrhagic and thrombotic diseases, may improve understanding hemostasis disorders in *Bothrops* envenomation. By combining rotational thromboelastometry (ROTEM), a point-of-care viscoelastic test of whole blood hemostasis allowing the global assessment of clot formation and its dissolution in real time [56], thrombin generation assay (TGA) assessing the balance between procoagulant and anticoagulant drivers [57] and fibrinography assessing fibrin clot formation and fibrinolysis [58], it is possible to overall investigate the complex time-course of venom-induced hypo- and hypercoagulability.

We therefore designed a rat model of human *B*. *atrox* and *B*. *lanceolatus* envenomation aiming to identify the determinants of the hemorrhagic and thrombotic effects of venoms and the contributions of the various key-players of hemostasis, *i*.*e*., platelets, coagulation factors, and endothelial cells.

## Material and methods

### Ethics statement

Our animal experiments complied with the ARRIVE guidelines and were carried out in accordance with the EU Directive 2010/63/EU for animal experiments and the ethical guidelines established by the National Institutes of Health. The experimental protocols were approved by Paris Cité University Animal Care Ethics Committee and the French Ministry of Research (N° APAFIS #26913-20200-120174-024616-V5).

### Venoms

Freeze-dried venoms were obtained from Latoxan (Valence, France). *B*. *atrox* venom (batch 211.191) is a pool of samples from wild-caught or born-in-captivity, male and female adult snakes, from French Guiana, Peru and Brazil, whereas *B*. *lanceolatus* venom (batch 411.171) is a pool of samples from wild-caught, two male and one female, adult snakes, from Martinique. Venoms were stored at +4°C and dissolved in saline before injection. The lethal dose-50% (LD_50_) of *B*. *lanceolatus* venom was previously determined at 6 mg/kg in mice by intraperitoneal route [42]. Since preliminary *in vitro* experiments conducted in our laboratory using ROTEM in the rat whole blood showed that the procoagulant effect of *B*. *atrox* venom was 2-fold that of *B*. *lanceolatus* venom, we used 220% of the estimated intraperitoneal LD_50_ in mice for *B*. *lanceolatus* venom (*i*.*e*., 4 mg *per* rat), and half of this value for *B*. *atrox* venom (*i*.*e*., 2 mg *per* rat) to target equipotent doses.

### Antivenoms

Inoserp South America (Inosan Biopharma, Mexico) is an experimental polyvalent antivenom against *B*. *lanceolatus*, *B*. *atrox*, *B*. *alternatus*, *B*. *asper*, *B*. *jararaca*, *B*. *jararacussu*, *B*. *diporus*, *B*. *schlegeii*, *Lachesis muta*, *L*. *melanocephala*, *L*. *stenophrys*, and *Crotalus simus*. This antivenom consists of soluble IgG F(ab’)_2_ fragments. Based on the potency of the supplied batch (0IT06007; expiry date, June 2022), 1 mL neutralizes 9.9 mg of *B*. *lanceolatus* venom and 2.7 mg of *B*. *atrox* venom, as stipulated by the manufacturer.

### Animals

We used male Sprague-Dawley rats (Janvier-labs, France), weighing 250–400 g, housed for 7 days prior to experiment, maintained under constant temperature conditions (19–21°C), and submitted to a 12h/12h light/dark cycle, with food and water provided *ad libitum*.

### In vitro procedure testing by rotational thromboelastometry non-treated rat whole blood spiked with increasing venom concentrations

The experiment consisted of testing the rat whole blood by ROTEM after adding different concentrations of venom or r ex-tem (recombinant tissue factor and phospholipids, #503–05, positive control) or saline (negative control). The triggering reagent was therefore either venom or r ex-tem while the addition of saline evaluates spontaneous coagulation.

Whole blood was sampled from the catheterized abdominal aorta in a non-treated rat anesthetized using 10mg/kg xylazine and 70 mg/kg ketamine injected intraperitoneally. Syringe pre-filled with 0.4 mL of buffered sodium citrate (final molarity: 0.109 M) was used to collect 3.6 mL of blood, then gently inverted five times to ensure adequate anticoagulation before transferring blood samples into tubes.

Rotational thromboelastometry was performed on ROTEM Delta analyzer (Werfen, Le Pré-Saint-Gervais, France) to assess the venom effect on coagulation system. For each venom, ROTEM was performed at five different venom concentrations (100, 10, 1; 0.1, and 0.01 μg/mL). Venom stock was diluted in saline phosphate buffer (PBS) to obtain a 3.5 mg/mL solution. The non-treated rat citrated whole blood tube was placed in the sample pre-heating station (temperature at 37°C) of the ROTEM analyzer. For the first venom concentration (100 μg/mL), all reagents were pipetted into the cup: 20 μL CaCl_2_ (Star-tem, #503–01), 20 μL venom sample, and 300 μL whole blood. Viscoelasticity data were then recorded at 37°C for 60 min. For the other venom concentrations, the volume of venom solution was adjusted to 20 μL using PBS. The positive control consisted on the same procedure using 20 μL of r ex-tem instead of venom solution, whereas the venom solution was replaced with 20 μL of PBS for the negative control (thus corresponding to the spontaneous coagulation activation in whole blood). Parameters assessed using ROTEM included clotting time (CT) and maximum clot firmness (MCF). CT is the time (s) from start of the measurement until initiation of clotting (i.e., clot firmness of 2 mm above base-line) and depends on the concentrations of coagulation factors. MCF is the maximum strength of the clot (mm) reached during the run, and depends on the platelet count and function and fibrin formation.

### Protocol design of the animal model of envenomation

Secondly, we developed an animal model of envenomation. For each condition (*B*. *atrox* venom, *B*. *lanceolatus* venom, and saline) and at each sampling time, rats were randomized (*n* = 6/group). Saline (control rat group) or venom (2 envenomated rat groups) was injected by the dorsal subcutaneous route, with an average 2 mL/kg volume. Blood was sampled at 3 h (H3), 6 h (H6), or 24 h (H24) after the saline/venom injection, from the catheterized abdominal aorta in a rat anesthetized using 10 mg/kg xylazine and 70 mg/kg ketamine injected intraperitoneally.

Syringes pre-filled with 400 μL of buffered sodium citrate (final molarity: 0.109 M) or 20 μL of EDTA were used to collect 3.6 mL and 2 mL of blood, respectively. They were gently inverted five times to ensure adequate anticoagulation then blood was transferred into dry tubes. Antivenom was added into the tubes (10 μL/mL blood) for venom antagonization before analysis.

ROTEM experiments were carried out immediately using citrated whole blood and platelet count was performed in EDTA whole blood up to 4 h after blood sampling. Citrate tubes were double-centrifuged at 2500g for 10 min, at room temperature without brake to obtain platelet-poor plasma. EDTA tubes were centrifuged at 1000g for 10 min, at room temperature. Citrated platelet-poor plasma and EDTA plasma were then aliquoted and stored at -80°C for analysis.

### Ex vivo procedure testing by rotational thromboelastometry venom-treated rat whole blood at different time-points

ROTEM experiments (EXTEM test) were conducted on citrated whole blood, according to the manufacturer’s instructions. The EXTEM test explores the tissue-factor coagulation pathway. Experiments were performed as previously described. In EXTEM test, 20 μL of Star-tem, 20 μL of r ex-tem and 300 μL whole blood were added into the cup. In addition to CT and MCF, the lysis index (LI) 30 was measured. LI30 is the residual clot firmness at 30 minutes from CT and reflects the fibrinolysis phase.

### Platelet count

Platelet count was performed in EDTA whole blood with a MS9-3 veterinary hematology analyzer (Melet Shloesing, Osny, France).

### Plasma fibrinogen concentrations

Fibrinogen level was measured on citrated platelet-poor plasma using the standard Clauss method. It was performed on STA-R Max automated coagulation analyzer system (Stago, Asnières sur Seine, France) using Dade Thrombin reagent (Siemens, Munich, Germany).

### Plasma von Willebrand Factor (VWF) antigen

VWF antigen was measured on citrated platelet-poor plasma using the ELISA Asserachrom VWF:Ag assay (Stago). Results were normalized using a normal rat citrated pool plasma.

### ADAMTS13 activity

ADAMTS13 activity was measured on citrated platelet-poor plasma by an in-house FRETS-VWF73 assay [59] using commercial recombinant FRETS-VWF73 peptide (Peptide Institute, Ibaraki, Japan). Results were normalized using a normal rat citrated pool plasma.

### Thrombin generation assay (TGA)

TGA was performed on ST Genesia analyzer system (Stago), a fully automated system enabling quantitative standardized TG assessment derived from Hemker’s fluorescence method, using dedicated reagents, calibrator and quality controls [60]. Thrombin generation is initiated by the addition of tissue factor (TF) and phospholipid vesicles. STG-ThrombiCal, a buffered solution containing a known fixed amount of human thrombin, is incubated in a cuvette with a solution containing a fixed concentration of Z-Gly-Gly-Arg-7-amino-4-methylcoumarin (AMC) fluorogenic substrate (STG-FluoSet) and calcium chloride (STG-FluoStart): the calibration curve is thus adjusted for the optical characteristics of the plasma sample correcting for the inner filter effect. Citrated platelet-poor plasma samples are run in a second cuvette with STG-ThromboScreen in the absence of thrombomodulin in parallel to STG-FluoSet. STG-ThromboScreen contains recombinant human TF, at an intermediate picomolar concentration. Four parameters were analyzed: lag time (min; time from test triggering to signal detection), time to peak (min; time necessary for thrombin concentration to reach its maximal value), peak height (PH; nmol/L; maximal thrombin concentration), and endogenous thrombin potential: ETP (nM·min; area under the thrombin time-concentration curve). Results are presented as absolute values.

### Fibrinography: fibrin polymerization assay and clot lysis study

Fibrin polymerization and fibrinolysis kinetics were assessed by turbidimetry in a TECAN Infinite M200Pro spectrophotometer (Tecan, Männedorf, Suisse) as previously described [58]. Briefly, 30 μL of imidazole buffer, 27 μL of citrated platelet-poor plasma, and 3 μL of recombinant tPA (Cryopep, Montpellier, France, final concentration: 250 ng/mL) were mixed, then 40 μL of this mixture was transferred in wells of a plate, where had been added 10 μL of a v/v mixture of FT (Innovin, Dade Behring, Marburg, Germany, final concentration: 1 pmol/L) and phospholipid vesicles (phospholipids TGT, Cryopep, Montpellier, France, final concentration: 4 μmol/L). Plasma clotting was triggered with 10 μL of N-2- hydroxyethylpiperazine-N′-2-ethanesulfonic acid (HEPES) 50 mmol/L containing 60 mg/mL bovine serum albumin, and 100 mmol/L CaCl_2_. Turbidimetry was recorded at 350 nm at 37°C every 6 s. Five parameters were analyzed: lag time (in seconds, time from test triggering to reach 15% of the maximal turbidity), time to peak (s; time necessary for turbidity to reach its maximal value), start tail (s; time necessary for turbidity to reach its minimal value) and peak height (PH; arbitrary units (AU); maximal turbidity value). Data were normalized for determining lag time, time to peak, and start tail.

### Plasma inflammatory and endothelial biomarkers (multiplex assays)

Multiplex assays using the Luminex Magpix platform (Luminex, Austin, TX, USA) were performed with the Rat Adipokine Magnetic Bead Panel (Merck Millipore, Molsheim, France) for the measurement of interleukin (IL)-1ß, IL-6, tumor necrosis factor alpha (TNF-α), monocyte chemoattractant protein-1 (MCP-1), plasminogen activator inhibitor type-1 (PAI-1), and the Rat Vascular Injury Magnetic Bead Panel for the measurement of soluble E-selectin and soluble intercellular adhesion molecule-1 (ICAM-1) in EDTA plasma. These kits were processed according to the manufacturer’s instructions.

### Statistical analysis

Analyses were performed using GraphPad PRISM 9.5.0 (GraphPad Prism Inc., La Jolia, CA, USA). All results are expressed as mean ± standard deviation for quantitative data and % of reference values for qualitative data. Data were tested for normality by visual inspection and Shapiro-Wilk tests. For *in vitro* experiments, ROTEM parameters had a normal distribution and were compared between the three experimental conditions (i.e., saline, *B*. *atrox* and *B*. *lanceolatus* venom) using one-way analysis of variance (ANOVA), followed by Tukey’s multiple tests. Distribution of measured values were represented as boxplots for CT and MCF. The median effective concentrations (EC_50_s) were determined visually using GraphPad PRISM 9.5.0.

For *ex vivo* experiments, parameters were compared between *B*. *atrox*, *B*. *lanceolatus*, and saline groups at each sample time using one-way ANOVAs (or a mixed-effect model in case of missing values) followed by Tukey’s multiple tests if parameters were normally distributed. If parameters were not normally distributed, comparisons were performed using Kruskal-Wallis’s tests followed by Dunn’s multiple tests. A *p*-value ≤ 0.05 was considered statistically significant.

## Results

### *In vitro* ROTEM assay: non-treated rat whole blood spiked with increasing venom concentrations

**Fig 1:**
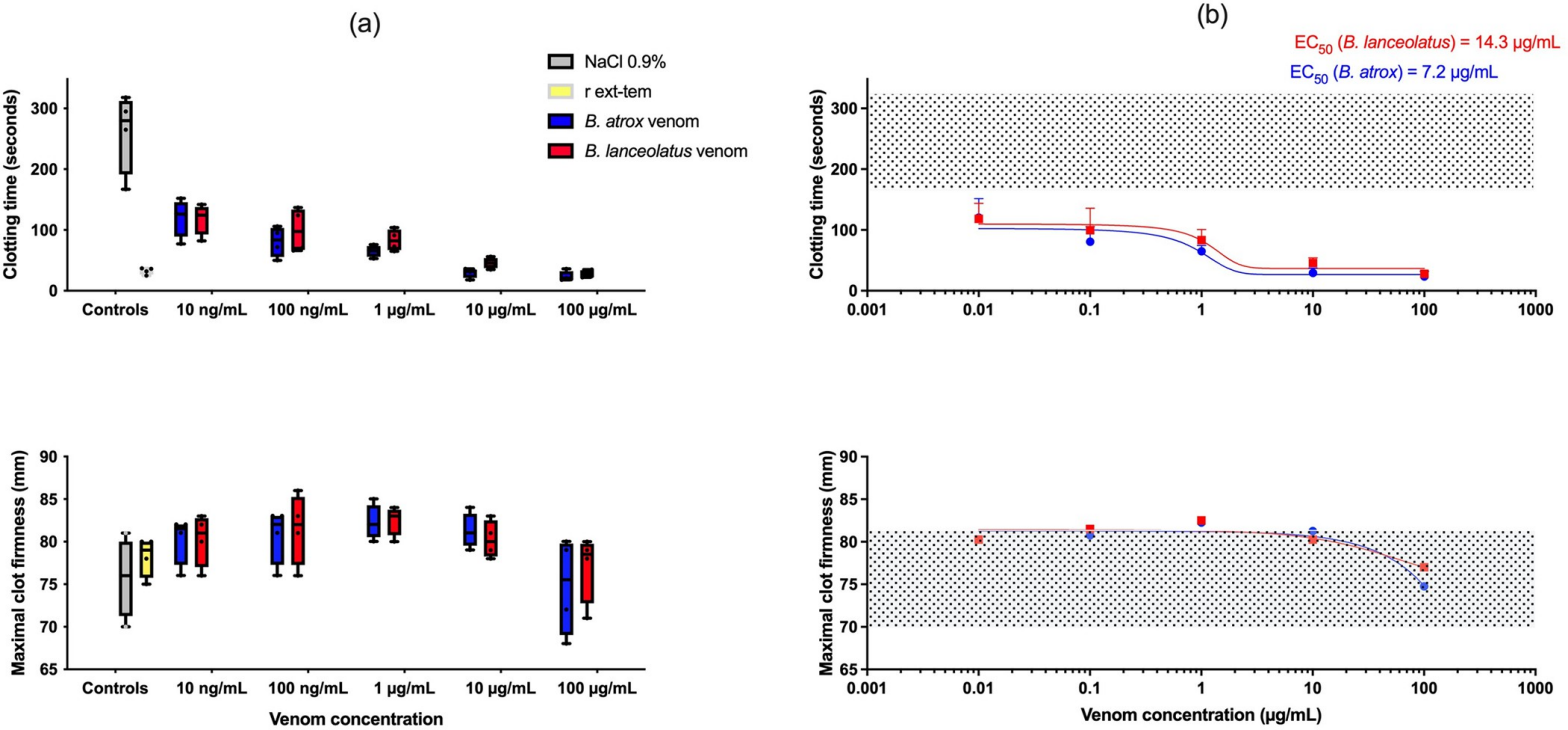
(a) Clotting time (CT) and maximal clot firmness (MCF) assessed by ROTEM in non-treated rat whole blood in presence of 0.9% NaCl (negative control, grey box-plot), r ex-tem (positive control, yellow box-plot), and *B*. *atrox* (blue box-plots) or *B*. *lanceolatus* venom (red box-plots) added at various concentrations (*n* = 4); (b) Curves representing the CT and the MCF as a function of the dose of *B*. *atrox* (red line) or *B*. *lanceolatus* (blue line) venom. Grey dotted area represents the value of negative control. Results are presented as mean ± SD (*n* = 4).

Negative control (rat whole blood with 0.9% NaCl) exhibited a CT of 261.2 ± 66.5 s and a MCF of 75.7 ± 4.6 mm. Positive control (with r ex-tem) exhibited a CT of 32.5 ± 5.5 s and a MCF of 78.2 ± 2.4 mm. *B*. *atrox* and *B*. *lanceolatus* venoms presented dose-dependent procoagulant activities with significant decrease in CT compared to the negative control (p = 0.0008) but no significant difference was observed when compared to one another (p = 0.1) (Figs 1A and S1). EC_50_ of *B*. *lanceolatus* venom was twice that of *B*. *atrox* venom (Fig 1B). In comparison to the negative control, *B*. *atrox* venom did not significantly alter MCF (p = 0.08) while *B*. *lanceolatus* venom showed a slight hypercoagulable profile (p = 0.02). There was no significant difference between the two venoms for MCF (p = 0.7) (Figs 1A and 1B and S1).

### *Ex vivo* ROTEM assay using venom-treated rat whole blood, platelet counts and fibrinogen at different time-points

**Fig 2:**
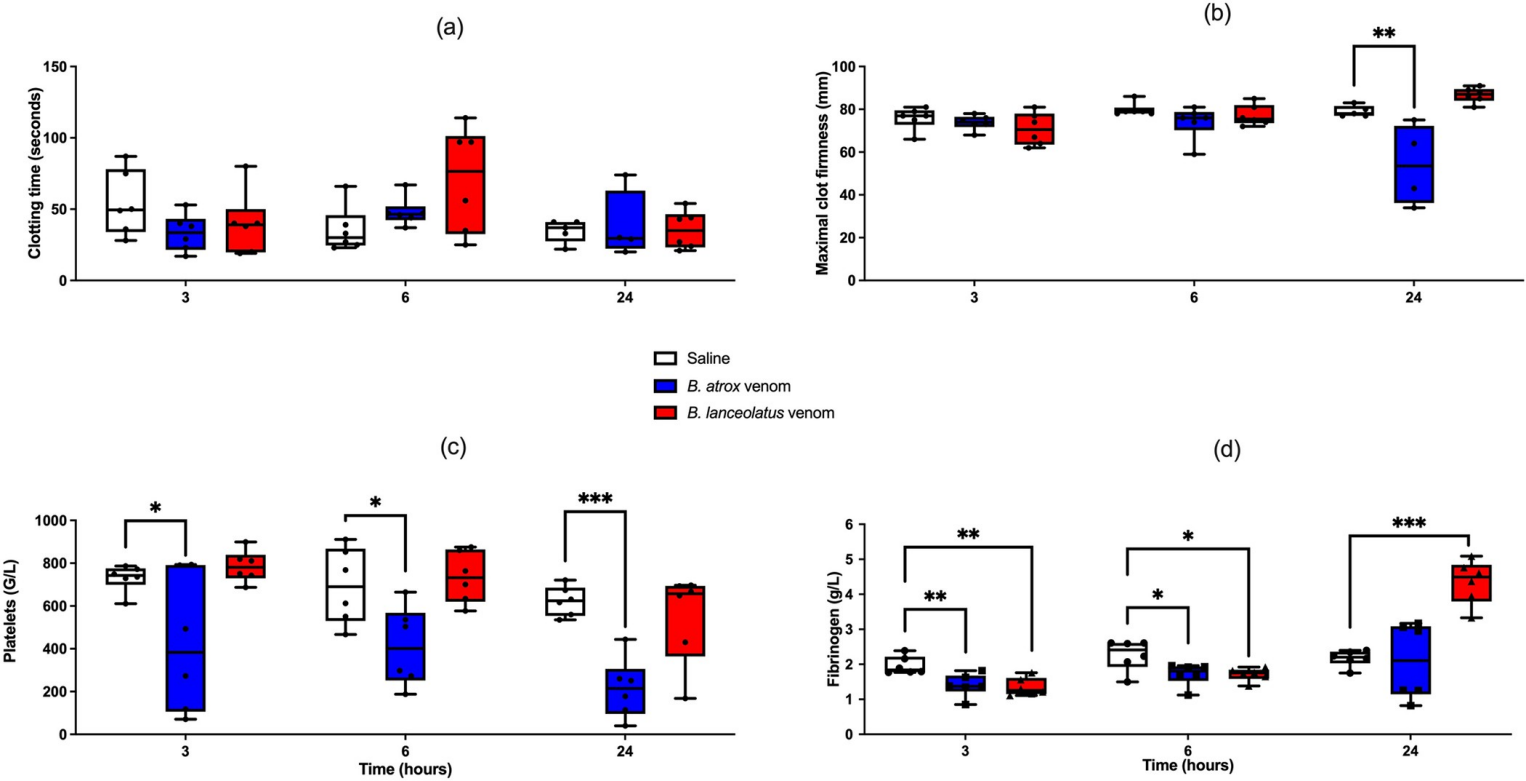
ROTEM clotting time (CT, a), ROTEM maximal clot firmness (MCF, b), platelet count (c), and plasma fibrinogen concentration (d) measured at H3, H6, and H24 after 0.9% NaCl (white boxplots), *B*. *atrox* (blue boxplots) or *B*. *lanceolatus* venom (red boxplots) injection in the rat. Results are presented as mean ± SD (*n* = 6 rats per group). *p < 0.05, **p < 0.01, ***p < 0.001 as compared to controls.

CT was not significantly modified at any time and for any venom (Figs 2A and S2). MCF of venom-treated rats did not differ from controls at H3 and H6. At H24, MCF of *B*. *atrox* venom-treated rats was significantly lower than controls (p = 0.004; Figs 2B and S2). No significant decrease in LI30 was observed whatever the time or the venom was. Significant thrombocytopenia was observed with *B*. *atrox* venom at H3 (p = 0.04), H6 (p = 0.02), and H24 (p < 0.001) while *B*. *lanceolatus* venom had no effect on platelet count (Fig 2C). Plasma fibrinogen was significantly lowered with the two venoms at H3 (p = 0.009 for *B*. *atrox* and p = 0.005 for *B*. *lanceolatus*) and at H6 (p = 0.03 for *B*. *atrox* and *B*. *lanceolatus*). At the opposite, plasma fibrinogen was significantly increased at H24 with *B*. *lanceolatus* venom (p < 0.001; Fig 2D).

### Thrombin generation assay (TGA)

**Fig 3:**
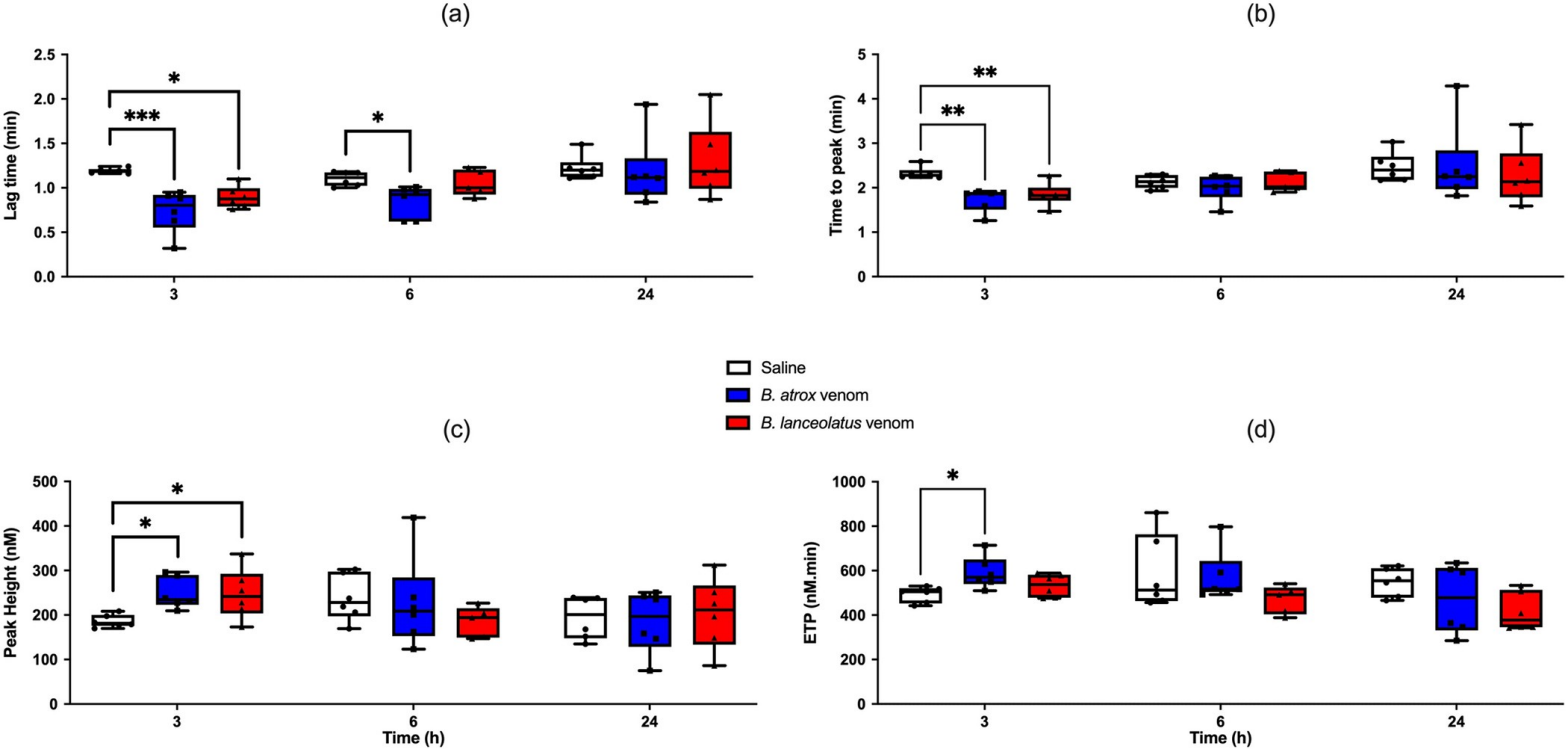
Lag time (a), time to peak (b), peak height (c), and endogen thrombin potential (ETP, d) assessed using thrombin generation assay (TGA) at H3, H6, and H24 after 0.9% NaCl (white boxplots), *B*. *atrox* (blue boxplots) or *B*. *lanceolatus* venom (red boxplots) injection in rats. Results are presented as mean ± SD (*n* = 6 rats per group). *p < 0.05, **p < 0.01, ***p < 0.001 as compared to controls.

Alterations in TGA occurred early, almost exclusively at H3. Lag time significantly decreased with both venoms at H3 (p < 0.001 for *B*. *atrox* and p = 0.01 for *B*. *lanceolatus*) and only for *B*. *atrox* at H6 (p = 0.019) (Fig 3A). Similarly, the time to peak significantly decreased with both venoms at H3 (p = 0.001 for *B*. *atrox* and p = 0.006 for *B*. *lanceolatus*) (Fig 3B). In parallel, the peak height significantly increased with the two venoms at H3 (p = 0.04) (Fig 3C). The endogenous thrombin potential only significantly increased with *B*. *atrox* venom at H3 (p = 0.02) (Fig 3D).

### Fibrinography

**Fig 4:**
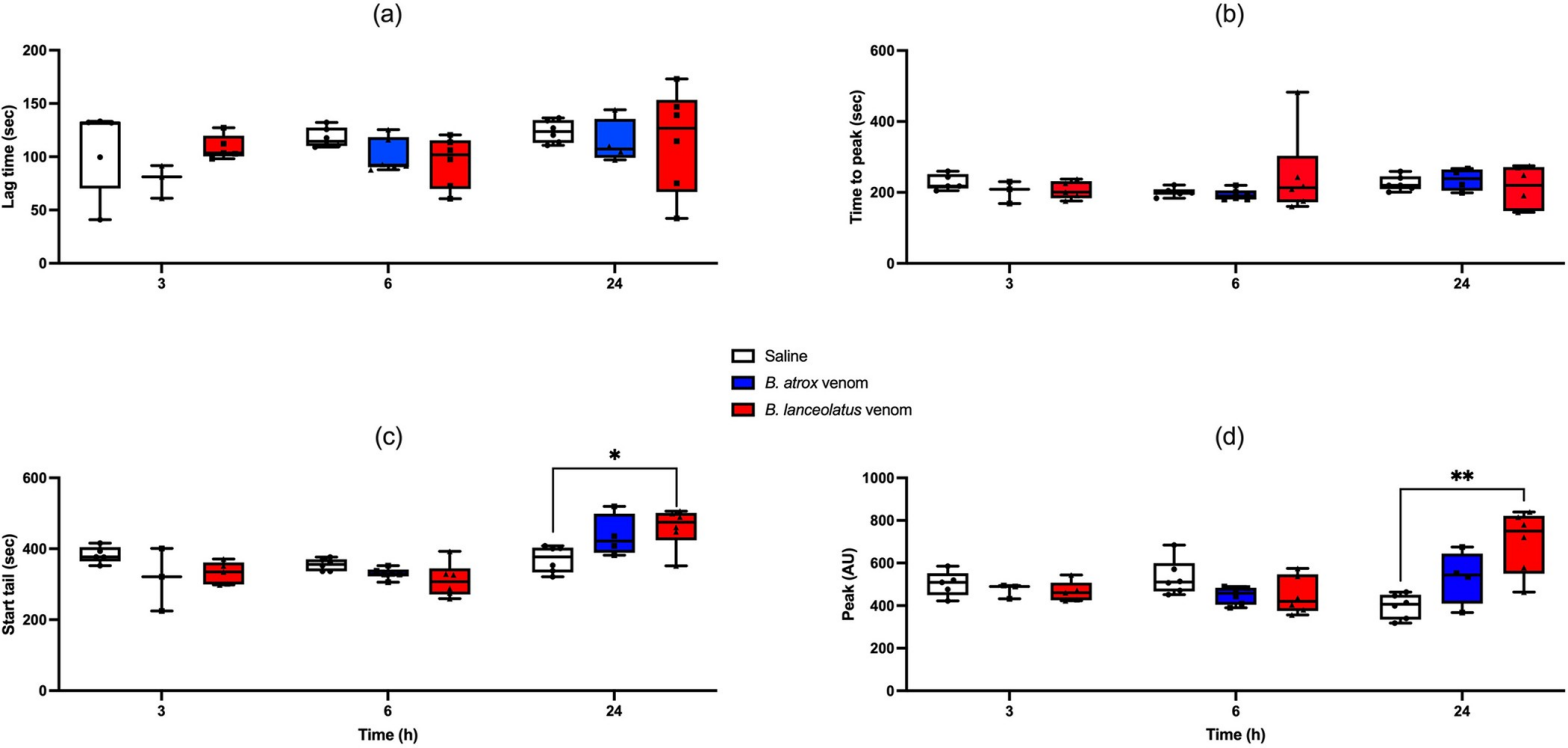
Lag time (a), time to peak (b), start tail (c), and peak height (d) assessed using fibrinography at H3, H6, and H24 after 0.9% NaCl (white boxplots), *B*. *atrox* (blue boxplots) or *B*. *lanceolatus* venom (red boxplots) injection in rats. Results are presented as mean ± SD (*n* = 6 rats per group). *p < 0.05, **p < 0.01 as compared to controls.

Both venoms did not significantly change the lag time and the time to peak of fibrin generation (Figs 4A and 4B and S3). *B*. *lanceolatus* venom significantly increased the start tail and the peak height at H24 (p = 0.03 and p = 0.002, respectively) (Figs 4C and 4D and S3).

### Endothelial function

**Fig 5:**
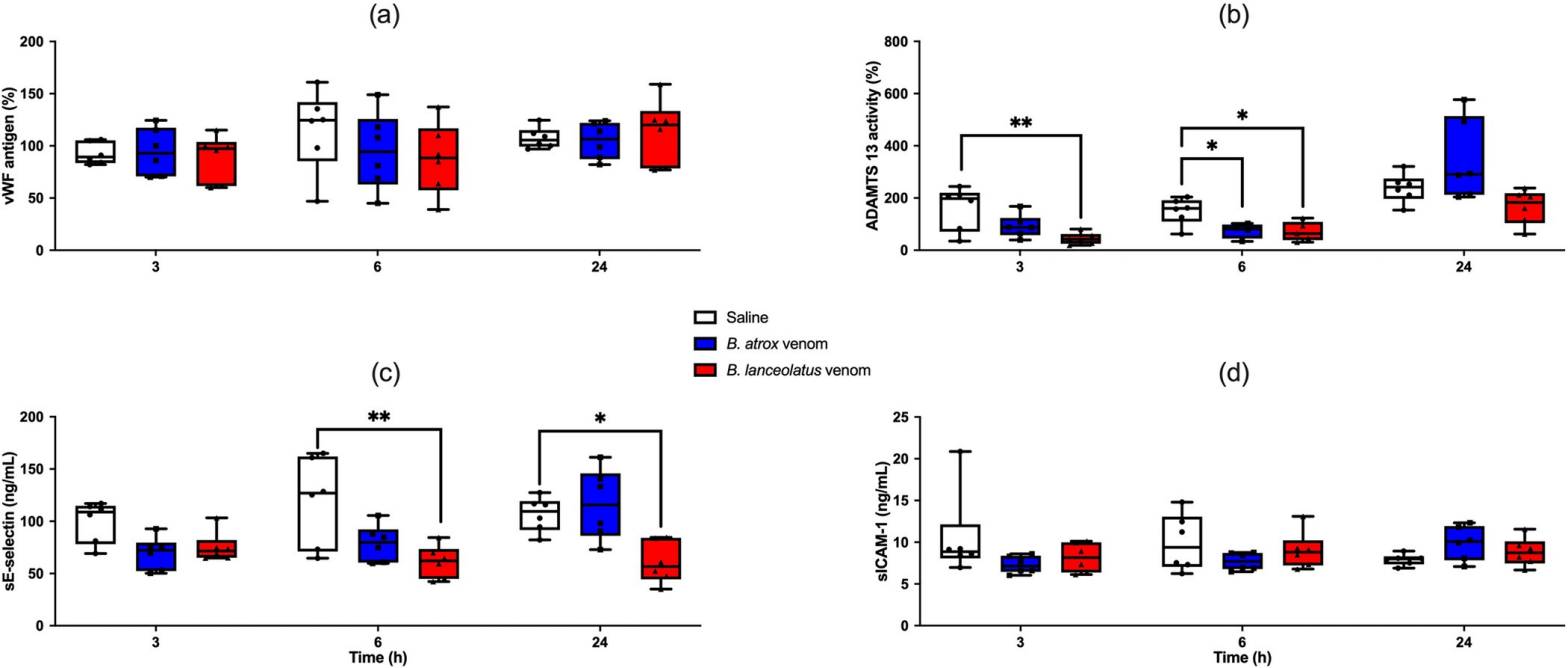
von Willebrand factor (VWF) antigen (a), ADAMTS13 activity (b), soluble E-selectin (c), and soluble intercellular adhesion molecule-1 (ICAM-1, d) measured at H3, H6, and H24 after 0.9% NaCl (white boxplots), *B*. *atrox* (blue boxplots) or *B*. *lanceolatus* venom (red boxplots) injection in rats. Results are presented as mean ± SD (*n* = 6 rats per group). *p < 0.05, **p < 0.01 as compared to controls.

No significant alteration was observed for plasma VWF antigen with any venom at any sampling time (Fig 5A). ADAMTS13 activity was significantly lowered at H3 with *B*. *lanceolatus* venom (p = 0.006) and at H6 with the two venoms (p = 0.04 for *B*. *atrox* and p = 0.02 for *B*. *lanceolatus*), before returning to non-significantly different values at H24 in comparison to the control group (Fig 5B). ADAMTS13 activity was not measured in 2 *B*. *atrox* venom-treated rats and one *B*. *lanceolatus*-treated rat due to insufficient sample volume. No modification in soluble E-selectin was observed whatever the venom at H3 and with *B*. *atrox* venom at H6 and H24 (Fig 5C). By contrast, a significant decrease in soluble E-selectin was observed with *B*. *lanceolatus* venom at H6 (p = 0.006) and H24 (p = 0.02). No modification in soluble ICAM-1 was observed whatever the venom or sampling time was (Fig 5D).

### Inflammatory parameters (multiplex assay)

**Fig 6:**
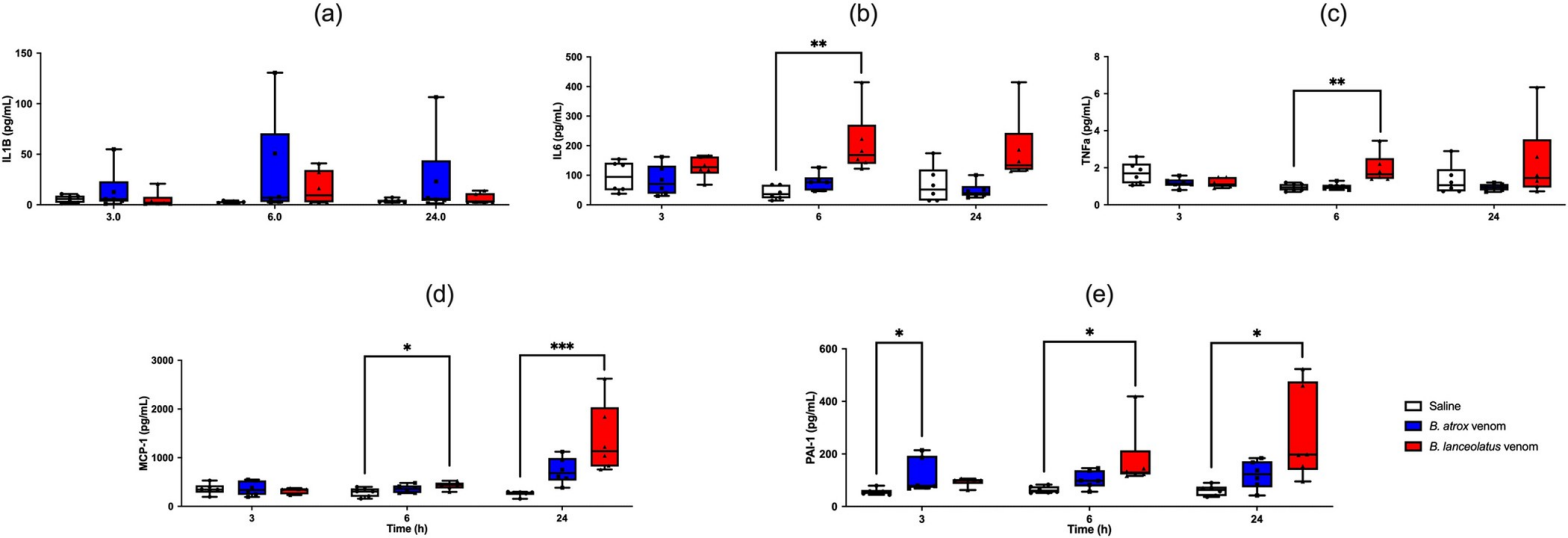
Plasma interleukin-1beta (IL-1β, a), interleukin-6 (IL-6, b) tumor necrosis factor-alpha (TNFα, c), plasminogen activator inhibitor-1 (PAI-1, d), and monocyte chemoattractant protein-1 (MCP-1, e) measured at H3, H6, and H24 after 0.9% NaCl (white boxplots), *B*. *atrox* (blue boxplots) or *B*. *lanceolatus* venom (red box-plots) injection in rats. Results are presented as mean ± SD (*n* = 6 rats per group). *p < 0.05, **p < 0.01, ***p < 0.001 as compared to controls.

We observed no significant modifications in plasma IL-1β (Fig 6A) but a transient increase in plasma IL-6 and TNF-α at H6 with *B*. *lanceolatus* venom (p = 0.001 and p = 0.007 respectively; Fig 6B and 6C), an increase in plasma MCP-1 at H6 (p = 0.031) and H24 (p < 0.001) with *B*. *lanceolatus* venom (Fig 6D) and an increase in PAI-1 at H3 with *B*. *atrox* venom (p = 0.049) and at H6 and H24 with *B*. *lanceolatus* venom (p = 0.039 and p = 0.01, respectively; Fig 6E).

## Discussion

Based on a global approach of hemostasis study, we determined the time-course of the common and differential impairments following *B*. *atrox* and *B*. *lanceolatus* envenomation in Sprague Dawley rats, with the aim of understanding the clinical complications observed in humans. In a previous study, we showed that *B*. *atrox* and *B*. *lanceolatus* venoms had a similar proteomic composition, with a predominance of SVMPs and a procoagulant effect on human whole blood [48]. Our *in vitro* results on venom-spiked rat whole blood were consistent with our previous findings, supporting procoagulant effects for both venoms.

Our *ex vivo* ROTEM investigation of venom-treated rat whole blood samples showed no reduction in CT at H3, suggesting venom-induced procoagulant effects of early and transient onset if any. Later, no prolongation of CT was recorded, suggesting no or only transient coagulation factor consumption undetectable at our sampling times. A previous work has reported a prolonged Extem CT at H1 after intravenous *B*. *asper* venom injection in mice which recovered at H3 [55]. A fast recovery of the clotting factors may correspond to the rapid elimination of venom procoagulant enzymes from the blood, consistent with reports on human snakebite envenomation in French Guiana in which prothrombin time and activated partial thromboplastin time were corrected faster than fibrinogen, even without antivenom administration [12,13]. Nevertheless, the hypothesis of lack of ROTEM sensitivity to identify any initial hypercoagulability in our animal model cannot be ruled out. Another hypothesis is that the dose of venom used was too low in this experiment.

Although not altered at H3 and H6, Extem MCF was significantly reduced at H24 after *B*. *atrox* venom injection, supporting the onset of venom-induced delayed hypocoagulability. This observation contrasted with Rucavado’s study reporting no clot at H1 after intravenous *B*. *asper* venom injection in mice but a steady increase in clot amplitude with decreased strength at H24 [55]. Noteworthy, here we used the subcutaneous route to mimic snakebite condition, which may account for the observed differences in fibrinogen kinetics. Based on our *in vitro* ROTEM study, no significant *B*. *atrox* venom-attributed effects were found except with the highest concentration tested. Whereas *ex vivo* MCF reduction is usually related to time-dependent venom enzymatic toxicity, *in vitro* reduction in MCF can be explained by platelet and fibrinogen consumption in a dose-dependent toxicity.

An early decrease in fibrinogen plasma level was observed with the two venoms at H3 and H6, whereas it recovered at H24 with *B*. *atrox* venom. Defibrinogenation occurs in most patients bitten by *B*. *atrox* [12,13,20,28,61]. A initial decrease in fibrinogen has also been reported in *B*. *lanceolatus* envenomation [38,39,62]. Hypofibrinogenemia is mostly due to the direct consumption induced by the TLE and fibrinogenases [7,37], but also possibly due to thrombin generation.

TGA showed a decrease in lag-time and time to peak and an increase in thrombin peak at H3 with the two venoms while ETP was only increased with *B*. *atrox* venom. TGA peak height is known to be more sensitive to variations in clotting factors than ETP [63]. Therefore, thrombin generation at H3 can be considered to be increased with both venoms. Interestingly, TGA was more sensitive than ROTEM to identify this initial hypercoagulability. To the best of our knowledge, this is the first study assessing thrombin generation in an animal model of *Bothrops* envenomation. TGA was already used to show that other Brazilian *Bothrops* venoms (i.e., *Bothrops moojeni*, *B*. *jararacussu*, and *B*. *alternatus*) increased endogenous thrombin potential of human platelet-poor plasma *in vitro* [35]. SVMP-induced intravascular thrombin generation plays a preponderant role in the pathogenesis of *B*. *jararaca*-related coagulopathy [52]. Decreased fibrinogen and prothrombin levels were significantly associated with systemic bleeding in *Bothrops* envenoming. The presence of such activators has been already reported with *B*. *atrox* [30–32] but never confirmed with *B*. *lanceolatus* venom yet despite *in vitro* data showing its ability to reduce coagulation time [45,46].

A gradual decrease in platelet count was only observed with *B*. *atrox* venom, starting at H3 and being maximal at H24. Because fibrinogen was corrected at that time, the decrease in ROTEM MCF at H24 was more related to platelet count impairment. Indeed, systemic bleeding has been attributed to thrombocytopenia in *B*. *atrox*-envenomated patients [28,64]. In *B*. *jararaca*-bitten patients, systemic bleeding is more frequent in the presence of acute thrombocytopenia than the blood hypocoagulability [65,66]. A negative correlation was found between platelet count and mean platelet volume in *B*. *atrox*-envenomated patients on admission, suggesting peripheral platelet destruction, which tends to increase the mean platelet volume [22]. Thrombocytin and batroxobin, two SVSPs isolated from *B*. *atrox* venom, induce washed human platelets aggregation in a less potent manner than thrombin [67,68]. Nevertheless, a direct activating effect of venom is unlikely: whole venom did not induce aggregation of washed rabbit platelets [69]. The increase in thrombin generation, which is considered as a powerful platelet activator, could be another explanation for thrombocytopenia [70]. However, this hypothesis seems unlikely since thrombin generation is increased with both venoms, whereas thrombocytopenia is only observed with *B*. *atrox* venom. Moreover, in a mouse model of *B*. *jararaca* envenomation, pretreatment with warfarin that decreases the prothrombin level thus the thrombin generation, did not prevent the occurrence of thrombocytopenia, suggesting a SVMP-independent mechanism. Finally, thrombocytopenia could be explained by platelet sequestration in the multiple bleeding sites caused by hemorrhagic SVMPs. Therefore, thrombocytopenia should be considered more as a consequence rather than a cause of venom-induced hemorrhages, although it contributes to bleeding.

A non-significant trend to MCF increase at H24 and a slight hypercoagulable profile were observed with *B*. *lanceolatus* venom in our rat model, nevertheless of little contribution to explain the mechanisms of thrombosis in this envenomation. Based on postmortem data in a *B*. *lanceolatus*-bitten patient, direct endothelial damage was hypothesized [47]. However, our results did not support this hypothesis. Despite a transient decrease in ADAMTS13 at H3 and H6, VWF antigen level was not increased after venom injection, which is consistent with previous findings in a model of *B*. *jararaca*-envenomated rat [53]. Partial recovery of plasma ADAMTS13 with Na_2_-EDTA suggested that its decrease was due to SVMPs and thrombin/plasmin generation [53]. Interestingly, an increase in VWF antigen level, not associated with an increase in tPA / PAI-1, was reported in *B*. *jararaca*-bitten patients, suggesting more endothelial activation by inflammation than a real endotheliopathy directly linked to the venom [65]. Here, we observed no increase in ICAM-1 nor soluble E-selectin levels, two biomarkers considered as more specific of endotheliopathy [71]. Consistently, *B*. *lanceolatus* venom did not increase ICAM-1 or E-selectin *in vitro* [72], supporting our hypothesis of a poor direct toxicity to endothelial cells. In a clinical study of *Bothrops* envenomations in Brazil, angiopoietin-1 and vascular cell adhesion protein-1 (VCAM-1) were only increased in acute kidney injury (AKI) patients whereas other biomarkers suggestive of endotheliopathy (i.e., syndecan-1, angiopoietin-2, vascular endothelial growth factor) were not significantly increased, even in AKI patients [73]. By contrast, elevated thrombomodulin levels were found in *B*. *jararaca*-bitten patients, suggesting endothelial damage at least of late-onset and/or in this snake species [74].

Venom-induced thrombotic complications could be explained, at least in part, by quantitative changes in fibrinogen. An increase in plasma fibrinogen at H24 was remarkable in our *B*. *lanceolatus* envenomation model. An increase in fibrinogen level during the follow-up and/or a late onset of hyperfibrinogenemia was often reported in patients with thrombosis in Martinique, also sometimes despite antivenom [39,47,62]. Early fibrinogen restoration leading to hyperfibrinogenemia might be explained by a less enzymatic activity of SVMPs isolated in *B*. *lanceolatus* venom, although considered as the most abundant family of proteins in this venom [45]. In mice, injection of *B*. *jararaca* venom upregulated hepatic mRNA synthesis of fibrinogen chains, which may explain the fast fibrinogen level recovery, even in the absence of antivenom [54]. In our model, fibrinography showed an increased fibrin generation at H24 which mechanism remains unexplained, but does not seem to be linked to a delayed thrombin generation, whose level was comparable to the control at H24. The increase in PAI-1 at H6 and H24 suggests an inhibition of the fibrinolytic pathway but ROTEM analysis may not show fibrinolysis shutdown. Perhaps *B*. *lanceolatus* venom is able to activate factor XIII: in *B*. *atrox* venom, thrombocytin is able to activate factor XIII by limited proteolysis and increase procoagulant activity of factor VIII similarly to thrombin [33]. Moreover, TLE from *B*. *lanceolatus* venom could increase fibrin generation: once bound to fibrin, the capacity of batroxobin, a TLE from *B*. *atrox* venom, to promote fibrin accretion was found 18-fold greater than that of thrombin [75].

Thrombosis may be due to coagulation-induced inflammatory activity of the venom. Here, we observed a systemic inflammatory response involving IL-6, TNF-α, and MCP-1 after *B*. *lanceolatus* venom injection. This result confirms an *ex vivo* model based on human whole blood, in which this venom elicited an inflammatory reaction combining pro-inflammatory interleukin production (IL-1β, IL-6 and TNF-α), chemokine upregulation (MCP-1, RANTES and IL-8), complement activation and eicosanoid release (leukotriene B_4_, prostaglandin E_2_ and thromboxane B_2_) [76]. These events were triggered by PLA_2_ isolated from *B*. *lanceolatus* venom [77]. In various other conditions including the coronavirus disease-2019, cytokine storm affects components of hemostasis, including endothelial cells, platelets, coagulation cascade, and fibrinolytic pathway, leading to hypercoagulability named thromboinflammation thus increasing the risk of thrombosis [78]. Consistent with the inflammatory response to venom, marked increase in C reactive protein (CRP) has been reported in *B*. *lanceolatus*-bitten patients [47,62]. However, thrombosis is not specific of *B*. *lanceolatus* envenomation but may rarely occur with *B*. *atrox* bite [79,80]. In an acute mesenteric ischemia case, mild coagulopathy associated with a marked inflammatory syndrome including hyperleukocytosis and increased CRP was described [80]. In another ischemic stroke case, limited alterations in hemostasis were reported on day 4 despite early antivenom administration [79]. Finally, TMA was diagnosed in several *Bothrops* envenomation patients, including by *B*. *jararaca* [81,82], *B*. *venezuelensis* [83] and *B*. *erythromelas* [84], presenting classical bothropic syndrome but no bleeding. Coagulopathy resolved within 12–24 hours after antivenom infusion, whereas TMA tended to start 1–3 days post-bite despite antivenom. Following *B*. *venezuelensis* bite, hyperfibrinogenemia began at day 2 and persisted over 2 weeks despite antivenom administration 4.5 h after the bite [83]. Here, we did not find a significant pro-inflammatory interleukin production with *B*. *atrox* venom despite an increasing trend in inflammatory biomarkers. Systemic inflammation has been demonstrated with *Bothrops* venoms [85,86]. An increase in IL-1β, IL-6 and MCP-1 but a decrease in TNF-α were observed in *B*. *atrox*-bitten patients [64,87]. No significant differences in proinflammatory cytokine levels were identified in hypofibrinogenemic compared to normal fibrinogen patients [87]. This excessive inflammatory response may have clinical consequences. Increased MCP-1 level was associated with a AKI in *B*. *atrox* envenomation in the Brazilian Amazon [88]. However, more studies are needed to identify inflammatory biomarkers predictive of the prothrombotic risk in the time-course of *Bothrops* envenomation, even after antivenom administration.

Our study has limitations. First, to understand the effects of *Bothrops* venoms on hemostasis, we used a rat model supporting envenomation features with this genus. However, extrapolation to humans should remain cautious, particularly for thrombin generation as peaks are twice lower in the rats than in humans [89]. Likewise, fibrinolysis in rats is slower than that reported in humans [90]. Secondly, we did not look for any bleeding or thrombosis in this model, consistent with the unsuccessful previous attempts to design a model of *B*. *lanceolatus* venom-induced thrombosis [41,91]. Insufficient blood samples for technical reasons prevented us to carry out all planned analyzes that might have improved our approach such as the study of the intrinsic pathway of coagulation using ROTEM with In-tem reagent. Moreover, our observation time was limited to the first 24 hours of envenomation, while in patients, thrombosis usually occurs 48 h after the bite, preventing us to investigate possible delayed-onset mechanisms such as endothelial damage. The procoagulant effect of *B*. *atrox* venom depends on various factors, notably the geographical origin of the specimens collected [58]. The venom, which we used, included venoms from French Guiana, Peru and Brazil, to represent possible region-dependent variability in venom properties. If we had used a venom from only one region, selective results might have been obtained. Finally, it would have been also interesting to test the same dose of *B*. *atrox* venom as that used for *B*. *lanceolatus*.

## Conclusion

**Fig 7:**
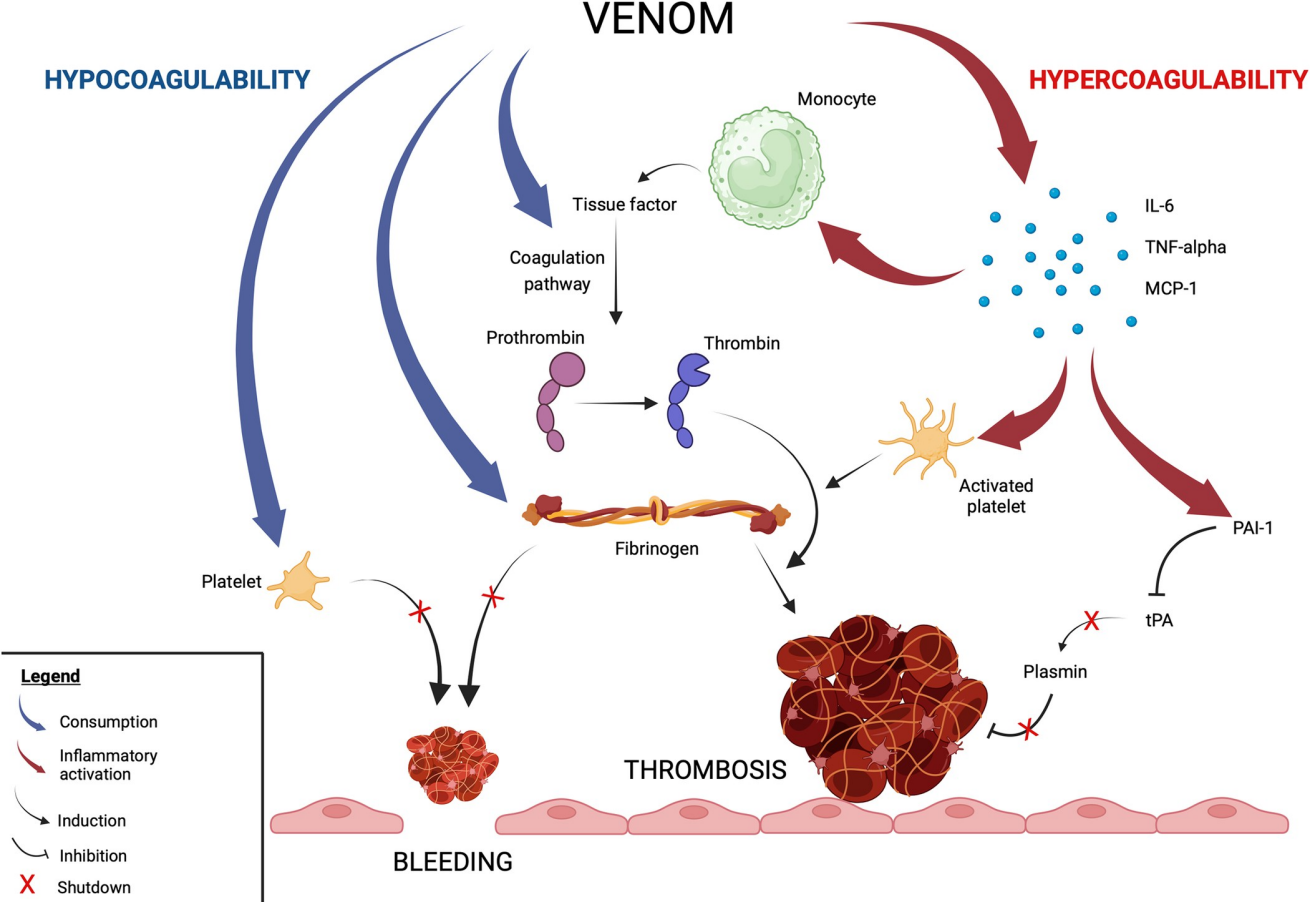
Hypothesized mechanisms of *Bothrops* venom-induced effects on hemostasis. The consumption of platelets and fibrinogen directly by the venom action and indirectly by coagulation factor activation induces a hypocoagulable state promoting bleeding. In contrast, proinflammatory cytokine (IL-6 and TNF-α) and chemokine production (MCP-1) increase tissue factor and factor XIII-A expression on monocytes, fibrinogen hepatocyte synthesis and release by activated platelets, and plasminogen activator inhibitor type I (PAI-1) production, which deactivates tissue plasminogen activator (tPA) and leads to plasmin decrease and fibrinolysis shutdown, inducing hypercoagulability and prothrombotic risk. Created with BioRender.com.

Our global investigation of hemostasis suggest that mechanisms involved in *Bothrops* envenomation-induced bleeding and thrombosis are not distinct but represent the two sides of the same coin (Fig 7). An initial step of venom-induced hypercoagulability with increased thrombin generation could be considered followed by a secondary step of hypocoagulability with *B*. *atrox* venom (as shown at H24 with ROTEM data) or persistent hypercoagulability with *B*. *lanceolatus* venom (as shown with fibrinography data), while these two venoms both have a similar proteomic composition with a predominance of SVMPs and procoagulant activity in vitro. By analogy to the Greek myth of Charybdis and Scylla, *Bothrops* envenomation may thus follow two opposite clinical expressions: in the event of major platelet and fibrinogen consumption promoting bleeding, hemorrhage will occur early, whereas in case of minor consumption or early restoration of platelet and fibrinogen limiting bleeding, venom-induced systemic inflammation will expose to thrombotic complications of delayed onset. Our experimental findings also help understanding why *B*. *atrox* envenomation could be complicated with thrombosis lately, as sometimes reported in patients with moderate initial consumption coagulopathy.

## Supporting information

S1 Fig Representative thromboelastometry trace with non-treated rat whole blood in presence of (a) *B*. *atrox* venom added at various concentrations: 100 μg/mL (red trace), 10 μg/mL (green trace), 1 μg/mL (blue trace), 100 ng/mL (pink trace); (b) *B*. *lanceolatus* venom added at various concentrations: 100 μg/mL (red trace), 10 μg/mL (green trace), 1 μg/mL (blue trace), 100 ng/mL (pink trace); (c) *B*. *atrox* venom at 10 ng/mL (blue trace), *B*. *lanceolatus* venom at 10 ng/mL (red trace), 0.9% NaCl (pink trace) and r ex-tem (green trace).
(TIF)

S2 Fig Representative thromboelastometry trace at H3 (a), H6 (b) and H24 (c) after 0.9% NaCl (green line), *B*. *atrox* (blue line) or *B*. *lanceolatus* venom (red line) injection in rats.
(TIF)

S3 Fig Representative turbidimetry curve generating by fibrinography at H3 (a), H6 (b) and H24 (c) after 0.9% NaCl (black line), *B*. *atrox* (blue line) or *B*. *lanceolatus* venom (red line) injection in rats.
(TIF)

S1 Table Excel spreadsheet containing, in separate sheets, the underlying numerical data for Figs 1, 2, 3, 4, 5 and 6. (XLSX)

## References

[1] Lasne D, Jude B, Susen S. From normal to pathological hemostasis. Can J Anaesth J Can Anesth. 2006;53: S2–11. doi: 10.1007/BF03022247 16766787

[2] Arnout J, Hoylaerts MF, Lijnen HR. Haemostasis. Handb Exp Pharmacol. 2006; 1–41. doi: 10.1007/3-540-36028-x_1 17001771

[3] Hoffman M, Monroe DM. A cell-based model of hemostasis. Thromb Haemost. 2001;85: 958–965. 11434702

[4] Levy JH, Szlam F, Tanaka KA, Sniecienski RM. Fibrinogen and hemostasis: a primary hemostatic target for the management of acquired bleeding. Anesth Analg. 2012;114: 261–274. doi: 10.1213/ANE.0b013e31822e1853 21965371

[5] Chapin JC, Hajjar KA. Fibrinolysis and the control of blood coagulation. Blood Rev. 2015;29: 17–24. doi: 10.1016/j.blre.2014.09.003 25294122 PMC4314363

[6] Furlan M, Robles R, Lämmle B. Partial purification and characterization of a protease from human plasma cleaving von Willebrand factor to fragments produced by in vivo proteolysis. Blood. 1996;87: 4223–4234. 8639781

[7] Berling I, Isbister GK. Hematologic effects and complications of snake envenoming. Transfus Med Rev. 2015;29: 82–89. doi: 10.1016/j.tmrv.2014.09.005 25556574

[8] Gutiérrez JM, Calvete JJ, Habib AG, Harrison RA, Williams DJ, Warrell DA. Snakebite envenoming. Nat Rev Dis Primer. 2017;3: 17063. doi: 10.1038/nrdp.2017.63 28905944

[9] Seifert SA, Armitage JO, Sanchez EE. Snake Envenomation. N Engl J Med. 2022;386: 68–78. doi: 10.1056/NEJMra2105228 34986287 PMC9854269

[10] Rautsaw RM, Jiménez-Velázquez G, Hofmann EP, Alencar LRV, Grünwald CI, Martins M, et al. VenomMaps: Updated species distribution maps and models for New World pitvipers (Viperidae: Crotalinae). Sci Data. 2022;9: 232. doi: 10.1038/s41597-022-01323-4 35614080 PMC9132920

[11] Larréché S, Chippaux J-P, Chevillard L, Mathé S, Résière D, Siguret V, et al. Bleeding and Thrombosis: Insights into Pathophysiology of Bothrops Venom-Related Hemostasis Disorders. Int J Mol Sci. 2021;22: 9643. doi: 10.3390/ijms22179643 34502548 PMC8431793

[12] Heckmann X, Lambert V, Mion G, Ehrhardt A, Marty C, Perotti F, et al. Failure of a Mexican antivenom on recovery from snakebite-related coagulopathy in French Guiana. Clin Toxicol Phila Pa. 2021;59: 193–199. doi: 10.1080/15563650.2020.1786108 32609546

[13] Resiere D, Houcke S, Pujo JM, Mayence C, Mathien C, NkontCho F, et al. Clinical Features and Management of Snakebite Envenoming in French Guiana. Toxins. 2020;12. doi: 10.3390/toxins12100662 33086750 PMC7589911

[14] Resiere D, Mégarbane B, Valentino R, Mehdaoui H, Thomas L. Bothrops lanceolatus bites: guidelines for severity assessment and emergent management. Toxins. 2010;2: 163–173. doi: 10.3390/toxins2010163 22069552 PMC3206616

[15] Carrasco PA, Koch C, Grazziotin FG, Venegas PJ, Chaparro JC, Scrocchi GJ, et al. Total-evidence phylogeny and evolutionary morphology of New World pitvipers (Serpentes: Viperidae: Crotalinae). Cladistics Int J Willi Hennig Soc. 2023;39: 71–100. doi: 10.1111/cla.12522 36701490

[16] Bucaretchi F, Herrera SR, Hyslop S, Baracat EC, Vieira RJ. Snakebites by Bothrops spp in children in Campinas, São Paulo, Brazil. Rev Inst Med Trop Sao Paulo. 2001;43: 329–333. doi: 10.1590/s0036-46652001000600006 11781603

[17] Otero-Patiño R. Epidemiological, clinical and therapeutic aspects of Bothrops asper bites. Toxicon Off J Int Soc Toxinology. 2009;54: 998–1011. doi: 10.1016/j.toxicon.2009.07.001 19591857

[18] Pardal PP de O, Souza SM, Monteiro MR de C da C, Fan HW, Cardoso JLC, França FOS, et al. Clinical trial of two antivenoms for the treatment of Bothrops and Lachesis bites in the north eastern Amazon region of Brazil. Trans R Soc Trop Med Hyg. 2004;98: 28–42. doi: 10.1016/s0035-9203(03)00005-1 14702836

[19] Pérez-Gómez AS, Monteiro WM, João GAP, Sousa JD de B, Safe IP, Damian MM, et al. Hemorrhagic stroke following viper bites and delayed antivenom administration: three case reports from the Western Brazilian Amazon. Rev Soc Bras Med Trop. 2019;52: e20190115. doi: 10.1590/0037-8682-0115-2019 31340373

[20] Silva de Oliveira S, Campos Alves E, Dos Santos Santos A, Freitas Nascimento E, Tavares Pereira JP, Mendonça da Silva I, et al. Bothrops snakebites in the Amazon: recovery from hemostatic disorders after Brazilian antivenom therapy. Clin Toxicol Phila Pa. 2020;58: 266–274. doi: 10.1080/15563650.2019.1634273 31264481

[21] da Silva Souza A, de Almeida Gonçalves Sachett J, Alcântara JA, Freire M, Alecrim M das GC, Lacerda M, et al. Snakebites as cause of deaths in the Western Brazilian Amazon: Why and who dies? Deaths from snakebites in the Amazon. Toxicon Off J Int Soc Toxinology. 2018;145: 15–24. doi: 10.1016/j.toxicon.2018.02.041 29490236

[22] Oliveira SS, Alves EC, Santos AS, Pereira JPT, Sarraff LKS, Nascimento EF, et al. Factors Associated with Systemic Bleeding in Bothrops Envenomation in a Tertiary Hospital in the Brazilian Amazon. Toxins. 2019;11. doi: 10.3390/toxins11010022 30621001 PMC6356762

[23] Franceschi A, Rucavado A, Mora N, Gutiérrez JM. Purification and characterization of BaH4, a hemorrhagic metalloproteinase from the venom of the snake Bothrops asper. Toxicon Off J Int Soc Toxinology. 2000;38: 63–77. doi: 10.1016/s0041-0101(99)00127-0 10669012

[24] Lomonte B, Gutiérrez JM, Borkow G, Ovadia M, Tarkowski A, Hanson LA. Activity of hemorrhagic metalloproteinase BaH-1 and myotoxin II from Bothrops asper snake venom on capillary endothelial cells in vitro. Toxicon Off J Int Soc Toxinology. 1994;32: 505–510. doi: 10.1016/0041-0101(94)90302-6 8053003

[25] Moreira L, Borkow G, Ovadia M, Gutiérrez JM. Pathological changes induced by BaH1, a hemorrhagic proteinase isolated from Bothrops asper (Terciopelo) snake venom, on mouse capillary blood vessels. Toxicon Off J Int Soc Toxinology. 1994;32: 976–987. doi: 10.1016/0041-0101(94)90376-x 7985202

[26] Rucavado A, Lomonte B, Ovadia M, Gutiérrez JM. Local tissue damage induced by BaP1, a metalloproteinase isolated from Bothrops asper (Terciopelo) snake venom. Exp Mol Pathol. 1995;63: 186–199. doi: 10.1006/exmp.1995.1042 9062552

[27] Gutiérrez JM, Núñez J, Escalante T, Rucavado A. Blood flow is required for rapid endothelial cell damage induced by a snake venom hemorrhagic metalloproteinase. Microvasc Res. 2006;71: 55–63. doi: 10.1016/j.mvr.2005.10.007 16337973

[28] S Oliveira S, C Alves E, S Santos A, F Nascimento E, T Pereira JP, M Silva I, et al. Bleeding Disorders in Bothrops atrox Envenomations in the Brazilian Amazon: Participation of Hemostatic Factors and the Impact of Tissue Factor. Toxins. 2020;12. doi: 10.3390/toxins12090554 32872404 PMC7551922

[29] Santoro ML, Sano-Martins IS, Fan HW, Cardoso JLC, Theakston RDG, Warrell DA, et al. Haematological evaluation of patients bitten by the jararaca, Bothrops jararaca, in Brazil. Toxicon Off J Int Soc Toxinology. 2008;51: 1440–1448. doi: 10.1016/j.toxicon.2008.03.018 18471839

[30] Hofmann H, Dumarey C, Bon C. Blood coagulation induced by Bothrops atrox venom: identification and properties of a factor X activator. Biochimie. 1983;65: 201–210. doi: 10.1016/s0300-9084(83)80085-6 6405812

[31] Hofmann H, Bon C. Blood coagulation induced by the venom of Bothrops atrox. 1. Identification, purification, and properties of a prothrombin activator. Biochemistry. 1987;26: 772–780. doi: 10.1021/bi00377a018 3552031

[32] Hofmann H, Bon C. Blood coagulation induced by the venom of Bothrops atrox. 2. Identification, purification, and properties of two factor X activators. Biochemistry. 1987;26: 780–787. doi: 10.1021/bi00377a019 3552032

[33] Niewiarowski S, Kirby EP, Brudzynski TM, Stocker K. Thrombocytin, a serine protease from Bothrops atrox venom. 2. Interaction with platelets and plasma-clotting factors. Biochemistry. 1979;18: 3570–3577. doi: 10.1021/bi00583a021 476069

[34] Rosing J, Govers-Riemslag JW, Yukelson L, Tans G. Factor V activation and inactivation by venom proteases. Haemostasis. 2001;31: 241–246. doi: 10.1159/000048069 11910191

[35] Duarte RCF, Rios DRA, Leite PM, Alves LC, Magalhães HPB, Carvalho M das G. Thrombin generation test for evaluating hemostatic effects of Brazilian snake venoms. Toxicon Off J Int Soc Toxinology. 2019;163: 36–43. doi: 10.1016/j.toxicon.2019.03.012 30880188

[36] Castro HC, Zingali RB, Albuquerque MG, Pujol-Luz M, Rodrigues CR. Snake venom thrombin-like enzymes: from reptilase to now. Cell Mol Life Sci CMLS. 2004;61: 843–856. doi: 10.1007/s00018-003-3325-z 15095007 PMC11138602

[37] Sanchez EF, Flores-Ortiz RJ, Alvarenga VG, Eble JA. Direct Fibrinolytic Snake Venom Metalloproteinases Affecting Hemostasis: Structural, Biochemical Features and Therapeutic Potential. Toxins. 2017;9: E392. doi: 10.3390/toxins9120392 29206190 PMC5744112

[38] Thomas L, Tyburn B, Ketterlé J, Biao T, Mehdaoui H, Moravie V, et al. Prognostic significance of clinical grading of patients envenomed by Bothrops lanceolatus in Martinique. Members of the Research Group on Snake Bite in Martinique. Trans R Soc Trop Med Hyg. 1998;92: 542–545. doi: 10.1016/s0035-9203(98)90907-5 9861375

[39] Thomas L, Tyburn B, Bucher B, Pecout F, Ketterle J, Rieux D, et al. Prevention of thromboses in human patients with Bothrops lanceolatus envenoming in Martinique: failure of anticoagulants and efficacy of a monospecific antivenom. Research Group on Snake Bites in Martinique. Am J Trop Med Hyg. 1995;52: 419–426. doi: 10.4269/ajtmh.1995.52.419 7771608

[40] Estrade G, Garnier D, Bernasconi F, Donatien Y. [Pulmonary embolism and disseminated intravascular coagulation after being bitten by a Bothrops lanceolatus snake. Apropos of a case]. Arch Mal Coeur Vaiss. 1989;82: 1903–1905. 2514645

[41] Gutiérrez JM, Sanz L, Escolano J, Fernández J, Lomonte B, Angulo Y, et al. Snake venomics of the Lesser Antillean pit vipers Bothrops caribbaeus and Bothrops lanceolatus: correlation with toxicological activities and immunoreactivity of a heterologous antivenom. J Proteome Res. 2008;7: 4396–4408. doi: 10.1021/pr8003826 18785768

[42] Resiere D, Arias AS, Villalta M, Rucavado A, Brouste Y, Cabié A, et al. Preclinical evaluation of the neutralizing ability of a monospecific antivenom for the treatment of envenomings by Bothrops lanceolatus in Martinique. Toxicon Off J Int Soc Toxinology. 2018;148: 50–55. doi: 10.1016/j.toxicon.2018.04.010 29654867

[43] Bogarín G, Romero M, Rojas G, Lutsch C, Casadamont M, Lang J, et al. Neutralization, by a monospecific Bothrops lanceolatus antivenom, of toxic activities induced by homologous and heterologous Bothírops snake venoms. Toxicon Off J Int Soc Toxinology. 1999;37: 551–557. doi: 10.1016/s0041-0101(98)00193-7 10080358

[44] Lôbo de Araújo A, Kamiguti A, Bon C. Coagulant and anticoagulant activities of Bothrops lanceolatus (Fer de lance) venom. Toxicon Off J Int Soc Toxinology. 2001;39: 371–375. doi: 10.1016/s0041-0101(00)00139-2 10978756

[45] Alsolaiss J, Alomran N, Hawkins L, Casewell NR. Commercial Antivenoms Exert Broad Paraspecific Immunological Binding and In Vitro Inhibition of Medically Important Bothrops Pit Viper Venoms. Toxins. 2022;15: 1. doi: 10.3390/toxins15010001 36668821 PMC9862972

[46] Bourke LA, Zdenek CN, Tanaka-Azevedo AM, Silveira GPM, Sant’Anna SS, Grego KF, et al. Clinical and Evolutionary Implications of Dynamic Coagulotoxicity Divergences in Bothrops (Lancehead Pit Viper) Venoms. Toxins. 2022;14: 297. doi: 10.3390/toxins14050297 35622544 PMC9148167

[47] Malbranque S, Piercecchi-Marti MD, Thomas L, Barbey C, Courcier D, Bucher B, et al. Fatal diffuse thrombotic microangiopathy after a bite by the “Fer-de-Lance” pit viper (Bothrops lanceolatus) of Martinique. Am J Trop Med Hyg. 2008;78: 856–861. 18541759

[48] Larréché S, Bousquet A, Chevillard L, Gahoual R, Jourdi G, Dupart A-L, et al. Bothrops atrox and Bothrops lanceolatus Venoms In Vitro Investigation: Composition, Procoagulant Effects, Co-Factor Dependency, and Correction Using Antivenoms. Toxins. 2023;15: 614. doi: 10.3390/toxins15100614 37888645 PMC10611193

[49] Kuch U, Mebs D, Gutiérrez JM, Freire A. Biochemical and biological characterization of Ecuadorian pitviper venoms (genera Bothriechis, Bothriopsis, Bothrops and Lachesis). Toxicon Off J Int Soc Toxinology. 1996;34: 714–717. doi: 10.1016/0041-0101(96)00016-5 8817816

[50] Rodrigues CFB, Zdenek CN, Bourke LA, Seneci L, Chowdhury A, Freitas-de-Sousa LA, et al. Clinical implications of ontogenetic differences in the coagulotoxic activity of Bothrops jararacussu venoms. Toxicol Lett. 2021;348: 59–72. doi: 10.1016/j.toxlet.2021.05.005 34044056

[51] Sousa LF, Zdenek CN, Dobson JS, Op den Brouw B, Coimbra F, Gillett A, et al. Coagulotoxicity of Bothrops (Lancehead Pit-Vipers) Venoms from Brazil: Differential Biochemistry and Antivenom Efficacy Resulting from Prey-Driven Venom Variation. Toxins. 2018;10: E411. doi: 10.3390/toxins10100411 30314373 PMC6215258

[52] Senise LV, Yamashita KM, Santoro ML. Bothrops jararaca envenomation: Pathogenesis of hemostatic disturbances and intravascular hemolysis. Exp Biol Med Maywood NJ. 2015;240: 1528–1536. doi: 10.1177/1535370215590818 26080462 PMC4935303

[53] Thomazini CM, Sachetto ATA, de Albuquerque CZ, de Moura Mattaraia VG, de Oliveira AK, Serrano SM de T, et al. Involvement of von Willebrand factor and botrocetin in the thrombocytopenia induced by Bothrops jararaca snake venom. PLoS Negl Trop Dis. 2021;15: e0009715. doi: 10.1371/journal.pntd.0009715 34478462 PMC8445451

[54] Sachetto ATA, Jensen JR, Santoro ML. Liver gene regulation of hemostasis-related factors is altered by experimental snake envenomation in mice. PLoS Negl Trop Dis. 2020;14: e0008379. doi: 10.1371/journal.pntd.0008379 32479494 PMC7289449

[55] Rucavado A, Chacón M, Villalobos D, Argüello I, Campos M, Guerrero G, et al. Coagulopathy induced by viperid snake venoms in a murine model: Comparison of standard coagulation tests and rotational thromboelastometry. Toxicon Off J Int Soc Toxinology. 2022;214: 121–129. doi: 10.1016/j.toxicon.2022.05.042 35644489

[56] Whiting D, DiNardo JA. TEG and ROTEM: technology and clinical applications. Am J Hematol. 2014;89: 228–232. doi: 10.1002/ajh.23599 24123050

[57] Tripodi A. Thrombin Generation Assay and Its Application in the Clinical Laboratory. Clin Chem. 2016;62: 699–707. doi: 10.1373/clinchem.2015.248625 26955824

[58] Amadio P, Porro B, Sandrini L, Fiorelli S, Bonomi A, Cavalca V, et al. Patho- physiological role of BDNF in fibrin clotting. Sci Rep. 2019;9: 389. doi: 10.1038/s41598-018-37117-1 30674980 PMC6344484

[59] Joly BS, Stepanian A, Leblanc T, Hajage D, Chambost H, Harambat J, et al. Child-onset and adolescent-onset acquired thrombotic thrombocytopenic purpura with severe ADAMTS13 deficiency: a cohort study of the French national registry for thrombotic microangiopathy. Lancet Haematol. 2016;3: e537–e546. doi: 10.1016/S2352-3026(16)30125-9 27720178

[60] Foulon-Pinto G, Jourdi G, Perrin J, Abdoul J, Paris G, Gouin-Thibault I, et al. Study of thrombin generation with St Genesia to evaluate xaban pharmacodynamics: Analytical performances over 18 months. Int J Lab Hematol. 2021;43: 821–830. doi: 10.1111/ijlh.13443 33369212

[61] Houcke S, Pujo JM, Vauquelin S, Lontsi Ngoula GR, Matheus S, NkontCho F, et al. Effect of the time to antivenom administration on recovery from snakebite envenoming-related coagulopathy in French Guiana. PLoS Negl Trop Dis. 2023;17: e0011242. doi: 10.1371/journal.pntd.0011242 37093856 PMC10159357

[62] Thomas L, Chausson N, Uzan J, Kaidomar S, Vignes R, Plumelle Y, et al. Thrombotic stroke following snake bites by the “Fer-de-Lance”Bothrops lanceolatus in Martinique despite antivenom treatment: a report of three recent cases. Toxicon Off J Int Soc Toxinology. 2006;48: 23–28. doi: 10.1016/j.toxicon.2006.04.007 16750232

[63] Duchemin J, Pan-Petesch B, Arnaud B, Blouch M-T, Abgrall J-F. Influence of coagulation factors and tissue factor concentration on the thrombin generation test in plasma. Thromb Haemost. 2008;99: 767–773. doi: 10.1160/TH07-09-0581 18392335

[64] Coelho KF, Neves JCF, Ibiapina HNS, Magalhães-Gama F, Barbosa FBA, Silva FS, et al. Exploring the Profile of Cell Populations and Soluble Immunological Mediators in Bothrops atrox Envenomations. Toxins. 2023;15: 196. doi: 10.3390/toxins15030196 36977086 PMC10051854

[65] Kamiguti AS, Cardoso JL, Theakston RD, Sano-Martins IS, Hutton RA, Rugman FP, et al. Coagulopathy and haemorrhage in human victims of Bothrops jararaca envenoming in Brazil. Toxicon Off J Int Soc Toxinology. 1991;29: 961–972. doi: 10.1016/0041-0101(91)90079-7 1949067

[66] Sano-Martins IS, Santoro ML, Castro SC, Fan HW, Cardoso JL, Theakston RD. Platelet aggregation in patients bitten by the Brazilian snake Bothrops jararaca. Thromb Res. 1997;87: 183–195. doi: 10.1016/s0049-3848(97)00118-7 9259109

[67] Niewiarowski S, Kirby EP, Stocker K. Throbocytin-a novel platelet activating enzyme from Bothrops atrox venom. Thromb Res. 1977;10: 863–869. doi: 10.1016/0049-3848(77)90144-x 882970

[68] Teng CM, Ko FN. Comparison of the platelet aggregation induced by three thrombin-like enzymes of snake venoms and thrombin. Thromb Haemost. 1988;59: 304–309. 3291184

[69] Francischetti IM, Castro HC, Zingali RB, Carlini CR, Guimarães JA. Bothrops sp. snake venoms: comparison of some biochemical and physicochemical properties and interference in platelet functions. Comp Biochem Physiol C Pharmacol Toxicol Endocrinol. 1998;119: 21–29. doi: 10.1016/s0742-8413(97)00163-1 9580495

[70] Jurk K, Kehrel BE. Platelets: physiology and biochemistry. Semin Thromb Hemost. 2005;31: 381–392. doi: 10.1055/s-2005-916671 16149014

[71] Xing K, Murthy S, Liles WC, Singh JM. Clinical utility of biomarkers of endothelial activation in sepsis—a systematic review. Crit Care Lond Engl. 2012;16: R7. doi: 10.1186/cc11145 22248019 PMC3396237

[72] Delafontaine M, Villas-Boas IM, Mathieu L, Josset P, Blomet J, Tambourgi DV. Enzymatic and Pro-Inflammatory Activities of Bothrops lanceolatus Venom: Relevance for Envenomation. Toxins. 2017;9: E244. doi: 10.3390/toxins9080244 28783135 PMC5577578

[73] Mota SMB, Albuquerque PLMM, Meneses GC, da Silva Junior GB, Martins AMC, De Francesco Daher E. Role of endothelial biomarkers in predicting acute kidney injury in Bothrops envenoming. Toxicol Lett. 2021;345: 61–66. doi: 10.1016/j.toxlet.2021.04.010 33872748

[74] Kamiguti AS, Rugman FP, Theakston RD, Franca FO, Ishii H, Hay CR. The role of venom haemorrhagin in spontaneous bleeding in Bothrops jararaca envenoming. Butantan Institute Antivenom Study Group. Thromb Haemost. 1992;67: 484–488. 1631797

[75] Vu TT, Stafford AR, Leslie BA, Kim PY, Fredenburgh JC, Weitz JI. Batroxobin binds fibrin with higher affinity and promotes clot expansion to a greater extent than thrombin. J Biol Chem. 2013;288: 16862–16871. doi: 10.1074/jbc.M113.464750 23612970 PMC3675619

[76] Silva de França F, Gabrili JJM, Mathieu L, Burgher F, Blomet J, Tambourgi DV. Bothrops lanceolatus snake (Fer-de-lance) venom triggers inflammatory mediators’ storm in human blood. Arch Toxicol. 2021;95: 1129–1138. doi: 10.1007/s00204-020-02959-0 33398417

[77] Gabrili JJM, Pidde G, Magnoli FC, Marques-Porto R, Villas-Boas IM, Squaiella-Baptistão CC, et al. New Insights into Immunopathology Associated to Bothrops lanceolatus Snake Envenomation: Focus on PLA2 Toxin. Int J Mol Sci. 2023;24: 9931. doi: 10.3390/ijms24129931 37373079 PMC10298673

[78] Wang J, Doran J. The Many Faces of Cytokine Release Syndrome-Related Coagulopathy. Clin Hematol Int. 2021;3: 3–12. doi: 10.2991/chi.k.210117.001 34595461 PMC8432322

[79] Cañas CA. Brainstem ischemic stroke after to Bothrops atrox snakebite. Toxicon Off J Int Soc Toxinology. 2016;120: 124–127. doi: 10.1016/j.toxicon.2016.08.005 27527269

[80] Galan LEB, Silva VS, Silva VS, Monte RC, Jati SR, Oliveira IS, et al. Acute mesenteric ischemia following lancehead snakebite: an unusual case report in the Northernmost Brazilian Amazon. Front Med. 2023;10. Available: https://www.frontiersin.org/articles/10.3389/fmed.2023.1197446 37425310 10.3389/fmed.2023.1197446 PMC10323676

[81] Bucaretchi F, Pimenta MMB, Borrasca-Fernandes CF, Prado CC, Capitani EMD, Hyslop S. Thrombotic microangiopathy following Bothrops jararaca snakebite: case report. Clin Toxicol Phila Pa. 2019;57: 294–299. doi: 10.1080/15563650.2018.1514621 30444155

[82] Malaque CMS, Duayer IF, Santoro ML. Acute kidney injury induced by thrombotic microangiopathy in two cases of Bothrops envenomation. Clin Toxicol Phila Pa. 2019;57: 213–216. doi: 10.1080/15563650.2018.1510129 30430871

[83] Fuchs J, Faber K, Tuchscherer DT, Tsakiris DA, Weiler S, Hofer KE. Bite by a juvenile Bothrops venezuelensis (Venezuelan lancehead) resulting in severe envenomation: A case report. Toxicon Off J Int Soc Toxinology. 2020;180: 39–42. doi: 10.1016/j.toxicon.2020.04.002 32289355

[84] Mota SMB, Albuquerque PLMM, Silva Júnior GB da, Daher EDF. Thrombotic microangiopathy due to Bothrops erythromelas: a case report in Northeast Brazil. Rev Inst Med Trop Sao Paulo. 2020;62: e53. doi: 10.1590/s1678-9946202062053 32725056 PMC7384591

[85] Leonel TB, Gabrili JJM, Squaiella-Baptistão CC, Woodruff TM, Lambris JD, Tambourgi DV. Bothrops jararaca Snake Venom Inflammation Induced in Human Whole Blood: Role of the Complement System. Front Immunol. 2022;13: 885223. doi: 10.3389/fimmu.2022.885223 35720304 PMC9201114

[86] Cavalcante JS, Borges da Silva WRG, de Oliveira LA, Brito IMC, Muller KS, J Vidal IS, et al. Blood plasma proteome alteration after local tissue damage induced by Bothrops erythromelas snake venom in mice. J Proteomics. 2022;269: 104742. doi: 10.1016/j.jprot.2022.104742 36174952

[87] Wellmann IAM, Ibiapina HNS, Sachett JAG, Sartim MA, Silva IM, Oliveira SS, et al. Correlating Fibrinogen Consumption and Profiles of Inflammatory Molecules in Human Envenomation’s by Bothrops atrox in the Brazilian Amazon. Front Immunol. 2020;11: 1874. doi: 10.3389/fimmu.2020.01874 32973773 PMC7468254

[88] Neves JCF, Ibiapina HNS, Magalhães-Gama F, Sachett JAG, Silva IM, Coelho KF, et al. CCL-2 and CXCL-8: Potential Prognostic Biomarkers of Acute Kidney Injury after a Bothrops atrox Snakebite. Mediators Inflamm. 2022;2022: 8285084. doi: 10.1155/2022/8285084 36117588 PMC9473908

[89] Tarandovskiy ID, Shin HKH, Baek JH, Karnaukhova E, Buehler PW. Interspecies comparison of simultaneous thrombin and plasmin generation. Sci Rep. 2020;10: 3885. doi: 10.1038/s41598-020-60436-1 32127577 PMC7054422

[90] Jankun J, Selman SH, Keck RW, Łysiak-Szydłowska W, Skrzypczak-Jankun E. Very long half-life plasminogen activator inhibitor type 1 reduces bleeding in a mouse model. BJU Int. 2010;105: 1469–1476. doi: 10.1111/j.1464-410X.2009.08962.x 19912209

[91] Herrera C, Rucavado A, Warrell DA, Gutiérrez JM. Systemic effects induced by the venom of the snake Bothrops caribbaeus in a murine model. Toxicon Off J Int Soc Toxinology. 2013;63: 19–31. doi: 10.1016/j.toxicon.2012.10.023 23159397

