# Supplementary figures and images for: Bothrops venom-induced hemostasis disorders in the rat: Between Scylla and Charybdis
Source: PLoS Negl Trop Dis. 2023 Nov 27;17(11):e0011786. doi: 10.1371/journal.pntd.0011786 (PMC10703418; doi:10.1371/journal.pntd.0011786)

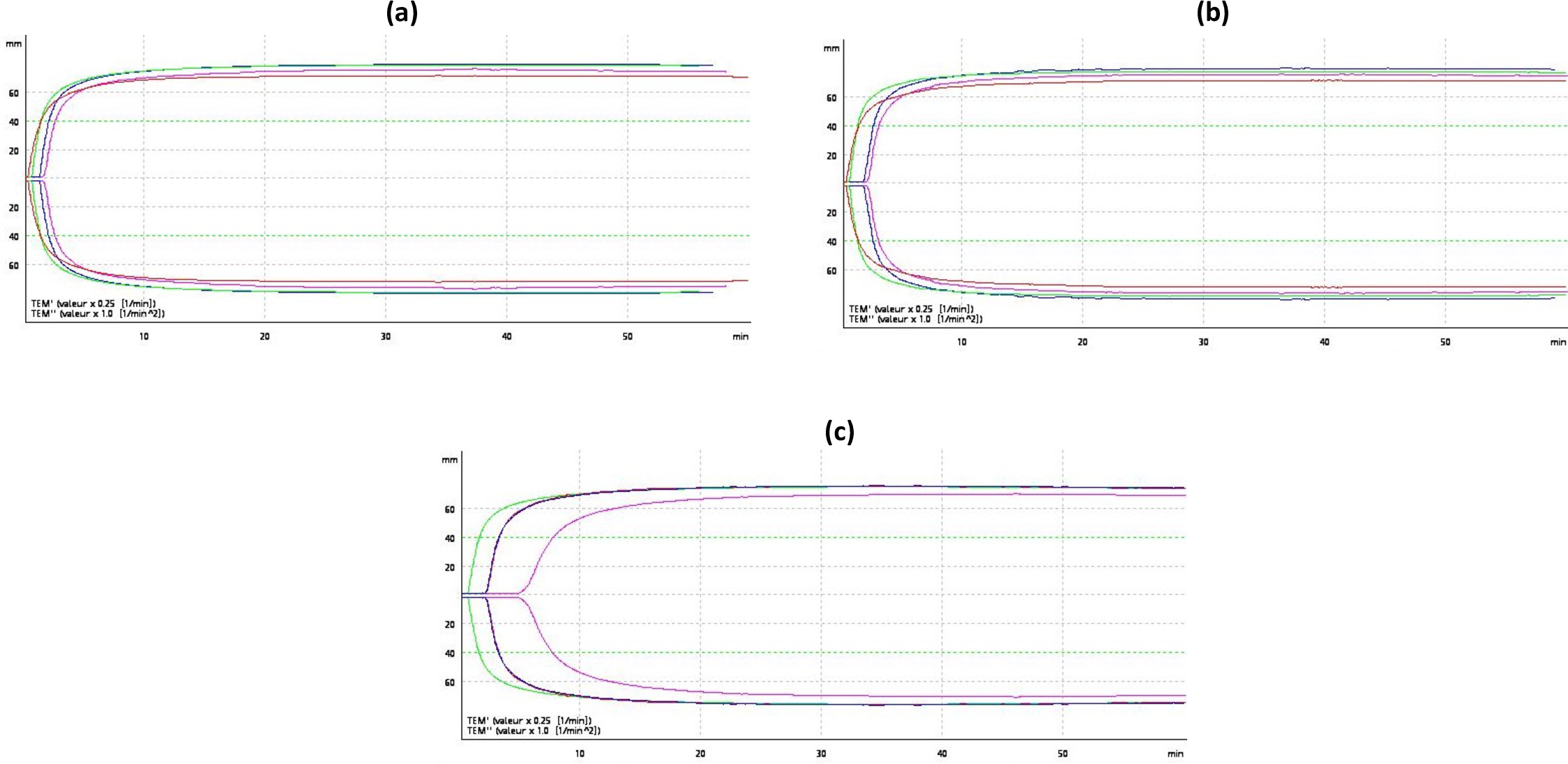

Supplement: S1 Fig — Representative thromboelastometry trace with non-treated rat whole blood in presence of (a) B. atrox venom added at various concentrations: 100 μg/mL (red trace), 10 μg/mL (green trace), 1 μg/mL (blue trace), 100 ng/mL (pink trace); (b) B. lanceolatus venom added at various concentrations: 100 μg/mL (red trace), 10 μg/mL (green trace), 1 μg/mL (blue trace), 100 ng/mL (pink trace); (c) B. atrox venom at 10 ng/mL (blue trace), B. lanceolatus venom at 10 ng/mL (red trace), 0.9% NaCl (pink trace) and r ex-tem (green trace). (TIF) [file pntd.0011786.s001.tif]

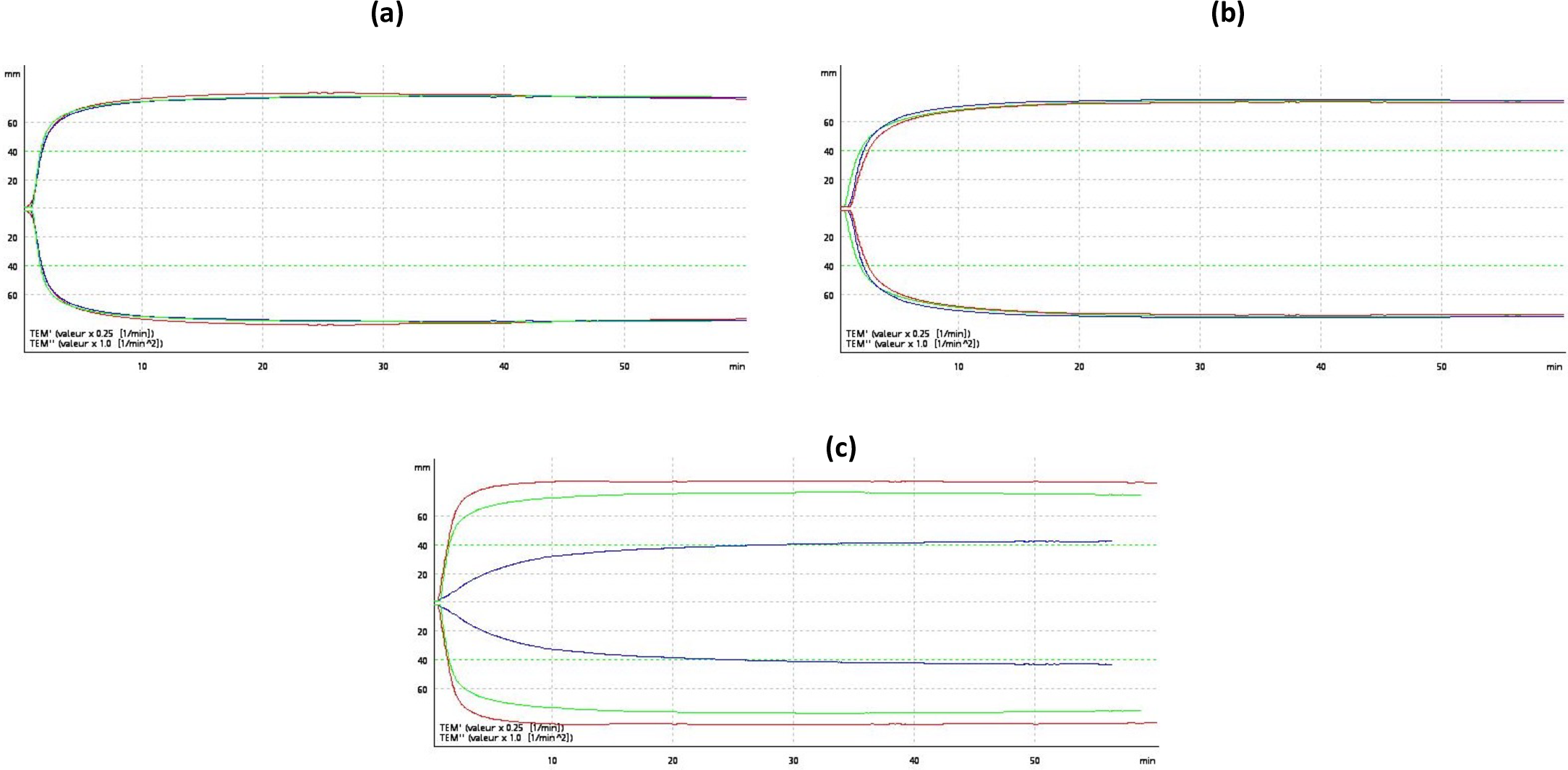

Supplement: S2 Fig — Representative thromboelastometry trace at H3 (a), H6 (b) and H24 (c) after 0.9% NaCl (green line), B. atrox (blue line) or B. lanceolatus venom (red line) injection in rats. (TIF) [file pntd.0011786.s002.tif]

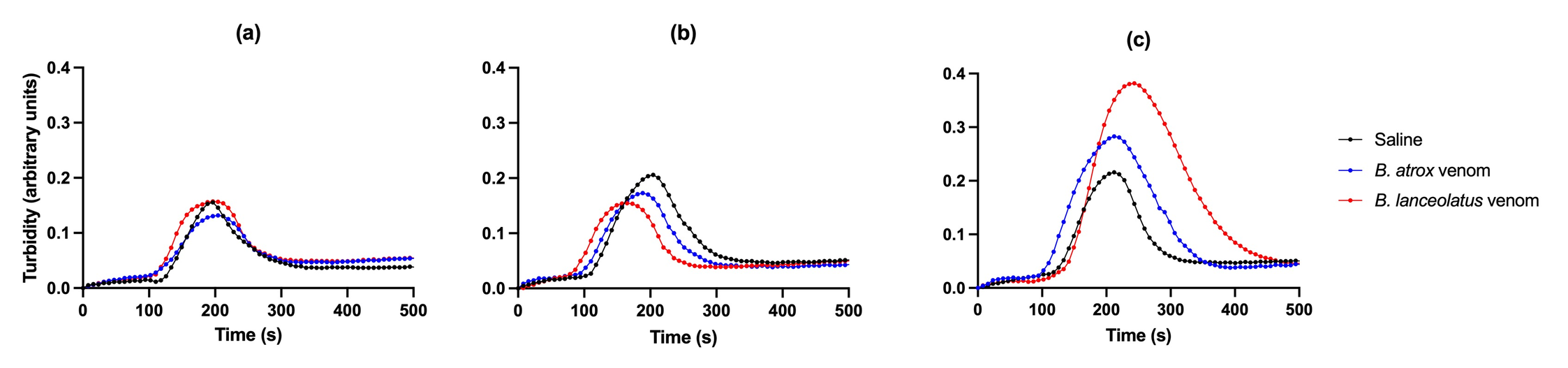

Supplement: S3 Fig — Representative turbidimetry curve generating by fibrinography at H3 (a), H6 (b) and H24 (c) after 0.9% NaCl (black line), B. atrox (blue line) or B. lanceolatus venom (red line) injection in rats. (TIF) [file pntd.0011786.s003.tif]
